# Antiglycative Properties of Anti-Dementia Drugs—In Vitro, In Silico Studies and a Systematic Literature Review

**DOI:** 10.3390/antiox14121509

**Published:** 2025-12-16

**Authors:** Wiktor Orlof, Jerzy Robert Ladny, Daniel Michalak, Małgorzata Zendzian-Piotrowska, Anna Zalewska, Mateusz Maciejczyk

**Affiliations:** 1 Department of Psychiatry, The Faculty of Medicine, Medical University of Bialystok, 2 Wolodyjowskiego Street, 15-272 Bialystok, Poland; w_orlof@wp.pl; 2 Department of Emergency Medicine and Disasters, Medical University of Bialystok, 37 Szpitalna Street, 15-767 Bialystok, Poland; ladnyjr@wp.pl; 3 Students Scientific Club “Biochemistry of Civilization Diseases”, Department of Hygiene, Epidemiology and Ergonomics, Medical University of Bialystok, 2c Mickiewicza Street, 15-233 Bialystok, Poland; dmichalak303@gmail.com; 4 Department of Hygiene, Epidemiology and Ergonomics, Medical University of Bialystok, 2c Mickiewicza Street, 15-233 Bialystok, Poland; malgorzata.zendzian-piotrowska@umb.edu.pl; 5 Experimental Dentistry Laboratory, Medical University of Bialystok, 24a M. Sklodowskiej-Curie Street, 15-274 Bialystok, Poland; azalewska426@gmail.com

**Keywords:** protein glycation, antiglycative activity, anti-dementia drugs, antiepileptic drugs, carbonyl stress

## Abstract

Protein glycation and oxidation contribute to the pathogenesis of neurodegenerative diseases. This study evaluated the antiglycative and antioxidative effects of donepezil, rivastigmine, galantamine, memantine, lamotrigine, sodium valproate, and carbamazepine using bovine serum albumin (BSA) as a model protein. Glycation was induced with fructose, ribose, or methylglyoxal (MGO), and oxidation with chloramine T (ChT). Concentrations of glycation products—Amadori products (APs), amyloid cross-β structure (βA), argpyrimidine (ARG), crossline (CRO), vesperlysine (VES), pentosidine (PEN), total AGEs and glycoxidation products—dityrosine (DT), kynurenine (KN), N-formylkynurenine (NFK) as well as oxidation biomarkers, total thiols (TTs), protein carbonyls (PCs), and advanced oxidation protein products (AOPPs), were determined via spectrophotometric and spectrofluorimetric methods. Molecular docking and a systematic literature review (PRISMA) complemented the experimental data. Lamotrigine showed the strongest antiglycative and antioxidative effects, surpassing aminoguanidine in reducing ARG, PEN, DT, and NFK levels. In contrast, donepezil markedly increased APs, βA, ARG, VES, DT, and PEN, suggesting proglycative and pro-oxidative activity. Docking revealed a high affinity of donepezil for RAGE (–7.2 kcal/mol), possibly explaining its impact on carbonyl stress. Overall, anti-dementia drugs showed weak to moderate antiglycative potential, with lamotrigine being the most effective.

## 1. Introduction

Neurodegenerative disorders, such as Alzheimer’s disease (AD), Parkinson’s disease (PD), amyotrophic lateral sclerosis (ALS), and Huntington’s disease (HD) pose a particular challenge for 21st century medicine [1]. They represent the leading cause of loss of cognitive ability, motor function, and independence, significantly reducing the quality of life of patients and their caregivers [2]. Epidemiological data indicate that dementia is the most common neurodegenerative disease [3,4]. AD currently affects between 15 and 21 million people worldwide [2]. The number of AD patients increases with age: The risk of developing the disease is 2% in people aged 60, 10% in those aged 65, and 50% in those aged 80. It is estimated that by 2050, approximately 139 million people will develop dementia [5]. The effective treatment of dementia is thus a priority for modern medicine. Current treatments only alleviate symptoms without stopping the progression of the disease [6]. Searching for new ways to diagnose, treat, and prevent dementia is one of the greatest challenges facing neurology, psychiatry, and translational medicine in the 21st century [7].

Protein glycation plays an important role in the pathogenesis of neuropsychiatric diseases [8,9,10]. Glycation is a nonenzymatic process that involves reducing sugars, such as glucose, fructose, and ribose, binding to free amino groups in proteins and lipids. This process has a significant impact on aging and numerous chronic diseases, including diabetes, atherosclerosis, and neurodegenerative disorders [11,12,13,14,15]. Glycation consists of three stages. Initially, the protein carbonyls of the sugar condense with the amino group of the protein, leading to the formation of an unstable Schiff base. Then, in an intermediate stage, the Schiff base is transformed into a more stable product called the Amadori product (APs). APs can undergo further reactions to form pentosidine (PEN) and vesperlysine (VES) [16]. In the final stage, advanced glycation end products (AGEs), such as crossline (CRO), are formed by oxidation, crosslinking, and protein polymerization reactions [17,18]. The interaction of AGEs with the receptor for advanced glycation end products (RAGE) contributes to organ damage, especially to the blood vessel walls and nerve cells [19,20]. These changes underlie many age-related diseases, including neuropsychiatric diseases [21,22]. Indeed, proteins altered by glycoxidation can weaken the blood–brain barrier, increasing its permeability [23]. It has been shown that AGEs derived from glyceraldehyde play a key role in promoting neuronal apoptosis [22]. Research also shows that oxidized amino acids, such as tryptophan (Trp) and tyrosine (Tyr), form toxic products during glycoxidation, including dityrosine (DT), kynurenine (KN), and N-formylkynurenine (NFK), which have neurotoxic effects [16,24,25]. Understanding the consequences of protein glycation has opened up new therapeutic prospects for treating neuropsychiatric diseases. Drugs and natural substances capable of inhibiting AGEs production and/or RAGE activation are being sought worldwide [26,27].

AD treatment focuses on slowing the progression of the disease, relieving symptoms, and improving patients’ quality of life [2]. In clinical practice, acetylcholinesterase inhibitors (AChEIs), such as donepezil, rivastigmine, and galantamine, which improve cognitive function, and the NMDA receptor antagonist memantine are used, especially in the moderate and severe disease stages [28]. In later stages of the disease, additional psychotropic medications, including antiepileptic drugs such as carbamazepine, lamotrigine, and valproic acid, may be introduced as adjunctive therapy to control behavioral and psychological symptoms of dementia (agitation, aggression, irritability, emotional instability) [29]. It is postulated that drugs used in AD therapy may exhibit additional mechanisms of action, including neurogenesis [30], neuroprotection [31], or antiglycative effects [14]. However, the available literature data are inconclusive. There is a lack of data on the antiglycative effect of anti-dementia drugs. Therefore, using various in vitro and in silico models, we were the first to compare the antiglycative activity of the most common drugs used to treat dementia. We also conducted a systematic literature review of the antiglycative properties of donepezil, rivastigmine, galantamine, memantine, lamotrigine, sodium valproate, and carbamazepine.

## 2. Materials and Methods

### 2.1. Reagents and Equipment

Donepezil, rivastigmine, and lamotrigine were purchased from AmBeed (3205N Wilke Rd, Ste3205-125, Arlington Hts, IL 60004, USA), galantamine and memantine from Sigma-Aldrich INC (2931 Soldier Springs Rd, Laramie, WY 82070, USA), sodium valproate at British Pharmacopoeia Commission Laboratory (Queens Rd, Teddington, Middlesex, TW11 OLY, UK), and carbamazepine at Sigma-Aldrich Co (3050 Spruce Street, St. Louis, MO 63101, USA) (Table 1). Bovine serum albumin (BSA) was sourced from Fisher BioReagents (Pittsburgh, PA, USA). The other reagents were purchased from Sigma-Aldrich (Numbrecht, Germany, and Saint Louis, MO, USA). All reagents were of analytical grade purity. Before testing, each solution was filtered using 0.2 mm membrane filters (Biosens, Warsaw, Poland). Fluorescence and absorbance were measured using an Infinite M200 PRO multimodal microplate reader (Tecan Group Ltd., Männedorf, Switzerland).

### 2.2. Bovine Serum Albumin (BSA)

High-purity BSA (over 98%, molar mass 66.430 Da) was dissolved in sodium phosphate buffer (0.1 M, pH 7.4) to obtain a concentration of 90 µmol/L, reflecting the physiological albumin content in the blood. Donepezil, rivastigmine, galantamine, memantine, lamotrigine, sodium valproate, and carbamazepine were then dissolved in BSA solution. The protein glycation inhibitor aminoguanidine and the well-known antioxidant N-acetylcysteine (NAC) were also used. All test substances were used at the same concentration of 1 mM. This concentration was set in proportion to the high levels of glycating/oxidizing agents in earlier in vitro kinetic experiments [18,32,33,34,35]. The entire study consisted of three independent experiments.

### 2.3. BSA Glycation Model

BSA glycation was carried out using the previous protocol [32,33,34]. Sugars, such as fructose, ribose, and aldehyde (methylglyoxal (MGO)), were used as the glycating agents.

The tested substances were incubated in a 90 µmol/L BSA solution with 0.5 M fructose and ribose for 10 days and with 2.5 mM MGO for 12 h. Samples were incubated in tightly sealed black plastic Falcon tubes, with constant stirring (50 rpm), in the dark, at 37 °C [32,33,34].

This model is commonly used to study the antiglycative properties of new substances. Although concentrations of glycating agents significantly exceed physiological levels in the human body, using higher concentrations is advantageous in the rapid modeling of biochemical processes that take weeks or even months in vivo [32,33,34].

To evaluate the rate of BSA glycation inhibition by the tested substances, the concentrations of the following were determined:

• glycation products: Amadori products (APs), amyloid cross-β structure (βA), argpyrimidine (ARG), crossline (CRO), vesperlysine (VES), pentosidine (PEN), and total AGEs,
• glyoxidation products: dityrosine (DT), kynurenine (KN), and N-formylkynurenine (NFK).

Colorimetric analysis was employed to determine the content of APs using nitro blue tetrazolium (NBT) reagent (Sigma-Aldrich; Saint Louis, MO, USA). Absorbance was measured at 525 nm. The monoformazan extinction coefficient of 12,640 M^−1^ cm^−1^ was used to calculate the APs content [36]. Fluorescence emitted during the binding of amyloid oligomers/fibrils to thioflavin T was used to estimate βA content. Fluorescence intensity was measured at 385/485 nm [37,38]. Fluorescent AGEs, such as ARG, CRO, VES, PEN, and total AGEs, were measured using fluorescence spectrophotometry. The study used excitation and emission wavelengths specific to analytes: 320/380 nm for ARG, 380/440 nm for CRO, 350/405 nm for VES, 335/385 nm for PEN, and an additional 350/440 nm for total AGEs. Before measurement, the analyzed samples were diluted with phosphate-buffered saline (PBS) at a ratio of 1:5 (*v*/*v*) [18,39,40].

Spectrofluorometric assays determined the level of protein glycoxidation products, including DT, KN, and NFK. Emission and excitation wavelengths specific to the analytes studied were 365/480 nm for DT, 330/415 nm for KN, and 325/434 nm for NFK. Before the spectrofluorimetric investigation, the samples were diluted with 0.1 M sulfuric acid (H_2_SO_4_) at a ratio of 1:5 (*v*/*v*) [39,41].

### 2.4. BSA Oxidation Model

BSA oxidation was carried out using the previous protocol [32,33,34]. Chloramine T (ChT) was introduced as an oxidizing agent.

The tested substances were incubated in a BSA solution with 20 mM ChT for 1 h. Samples were incubated in tightly sealed black plastic Falcon tubes, with constant stirring (50 rpm), in the dark, at 37 °C [32,33,34].

To evaluate the rate of BSA oxidation inhibition by the tested substances, the concentrations of the following were determined: total thiols (TTs), protein carbonyls (PCs), and advanced oxidation protein products (AOPPs).

The TTs concentration was determined by spectrophotometry at 412 nm using Ellman’s reagent (Sigma-Aldrich; Saint Louis, MO, USA) in 0.1 M phosphate buffer. The TTs content was determined based on the standard curve derived from reduced glutathione (GSH) [42]. A reaction with 2,4-dinitrophenylhydrazine (2,4-DNPH) determined the PCs concentration. Colorimetry was employed to establish the absorbance of the colored reaction products. The wavelength used in the test was 355 nm. The absorption coefficient for 2,4-DNPH of 22,000 M^−1^ cm^−1^ was used to calculate the concentration of PCs [18,43]. Spectrophotometry using potassium iodide and acetic acid determined the concentration of AOPPs. The absorbance of the samples was measured immediately after mixing the reagents at 340 nm [18,44].

### 2.5. Statistical Analysis

The study data were statistically analyzed using GraphPad Prism 9.000 for MacOS (GraphPad Software, Inc., La Jolla, CA, USA). Statistical significance compared to the control (BSA + glycating agent) was investigated using analysis of variance (ANOVA) with Dunett’s post hoc test. An adjusted *p*-value was calculated. Results are presented as mean ± standard deviation. A *p*-value of less than 0.05 was considered statistically significant.

### 2.6. Molecular Docking Analysis

The BSA crystal structure (PDB ID: 4F5S; 2.47 Å resolution) was downloaded from the Protein Data Bank in .pdb format [45]. Ligand structures were obtained from the National Library of Medicine in .sdf format [46]. All molecular structures were prepared in AutoDock MGL Tools (https://autodock.scripps.edu) by removing water molecules, adding polar hydrogens and Kollmann charges, and converting them to the .pdbqt format compatible with AutoDock Vina. Docking simulations were carried out in a 40 × 40 × 40 grid with 0.375 Å spacing, centered at coordinates 34.885, 23.976, and 98.792, with the exhaustiveness parameter set to 8 to improve the reliability of the binding pose and affinity predictions. Docking results were analyzed and visualized in PyMOL 2.5 to inspect ligand–protein interactions (e.g., hydrogen bonds, polar contacts, and hydrophobic interactions) and to calculate binding free energies. These in silico data were subsequently integrated with the in vitro findings to interpret the potential antiglycative or proglycative profiles of the tested drugs (see Section 3.3) [47,48].

### 2.7. Systematic Literature Review

The literature analysis covered the period from 1992 to January 2025 and was carried out using the Medline (PubMed) database. The available literature was analyzed using the following keywords: [name of drug* and antiglycoxidative properties], [name of drug* and antiglycative properties], [name of drug* and antiglycative properties], [name of drug* and glycation], [name of drug* and advanced glycation end products], [name of drug* and protein glycation]. The search included the following drugs*: donepezil, rivastigmine, galantamine, memantine, lamotrigine, sodium valproate, and carbamazepine. The inclusion and exclusion criteria applied to the publications analyzed are shown in Table 2.

**Figure 1:**
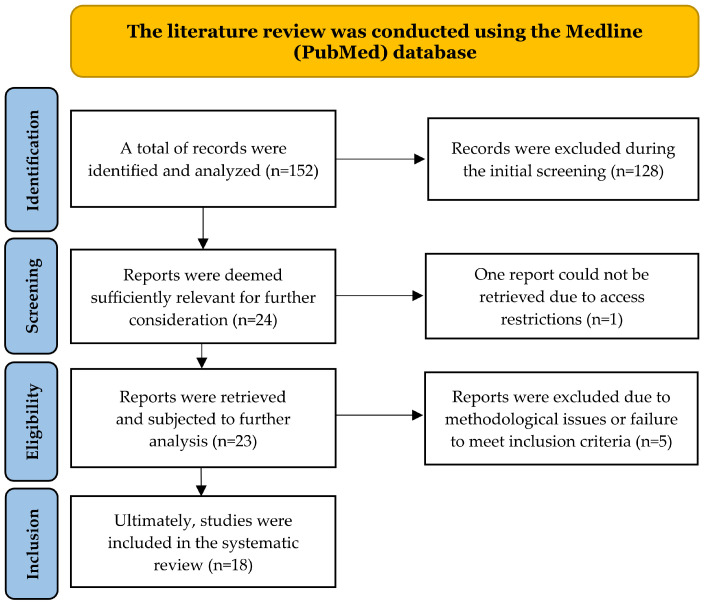
PRISMA flow diagram summarizing the study selection process. Records identified (n = 152), screened (n = 24), eligible (n = 23), and included (n = 18) are shown, with exclusions detailed at each stage.

The initial literature analysis involved two researchers (W.O., D.M.) independently reviewing publication titles and abstracts. Another pair of researchers (M.Z.P, A.Z.) then re-evaluated all previously shortlisted manuscripts. Eighteen studies were included in the final analysis based on the defined inclusion and exclusion criteria (Figure 1). Cohen’s kappa (κ) was calculated to determine the concordance between the researchers, yielding κ = 0.9. Each publication was assessed methodologically, with specific attention given to the authorship, publication year, study design, sample size, inclusion and exclusion criteria, research duration, and end points.

## 3. Results

### 3.1. BSA Glycation Model

We used a BSA glycation model to screen the antiglycative and antiglycoxidative potential of the selected drugs using three glycating factors (fructose, ribose, and MGO). BSA alone served as the negative control, whereas BSA incubated with each glycating agent (BSA + fructose, BSA + ribose, BSA + MGO) was used as the positive control. The drugs and aminoguanidine were added to BSA + glycating agent samples to assess whether they attenuate or potentiate the formation of glycation (APs, βA, ARG, CRO, VES, PEN, total AGEs) and glycoxidation markers (DT, KN, NFK) compared with the positive control.

#### 3.1.1. Fructose-Induced Glycation

Fructose induces protein glycation and crosslinking faster than glucose, mainly because it binds to proteins via carbon 2 (C-2), whereas glucose binds mainly via carbon 1 (C-1) [49]. This specific binding may account for the stronger glycation potential of fructose. In vitro studies have also shown that fructose generates significant amounts of glyoxal (GO) and MGO derivatives, key precursors of AGEs [50].

**Figure 2:**
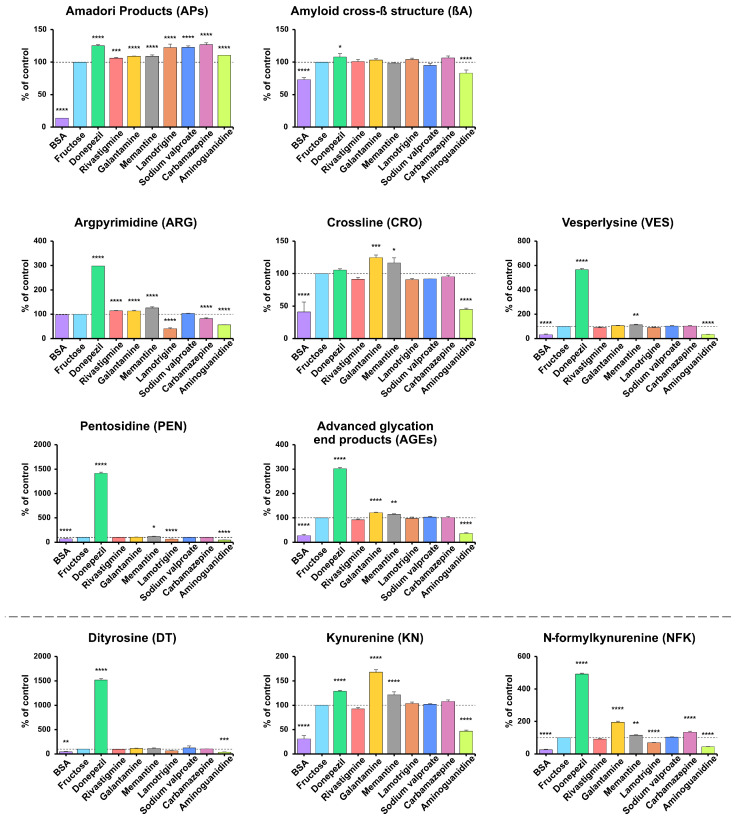
The inhibitory effects of donepezil, rivastigmine, galantamine, memantine, lamotrigine, sodium valproate, carbamazepine, and aminoguanidine on protein glycation and glycoxidation in fructose-induced glycation. APs, Amadori products; βA, amyloid cross-β structure; ARG, argpyrimidine; CRO, crossline; DT, dityrosine; KN, kynurenine; NFK, N-formylkynurenine; PEN, pentosidine; AGEs, advanced glycation end products; VES, vesperlysine; BSA, bovine serum albumin; fructose, albumin glycation induced by fructose; * *p* < 0.05 vs. positive control (glycating agent); ** *p* < 0.01 vs. positive control (glycating agent); *** *p* < 0.001 vs. positive control (glycating agent); **** *p* < 0.0001 vs. positive control (glycating agent).

The AP level was significantly lower in BSA compared to BSA incubated with fructose (–86.21%; *p* < 0.0001). Versus the positive control, the AP level was statistically higher in all samples: carbamazepine (+26.72%), donepezil (+24.87%), sodium valproate (+22.76%), lamotrigine (+22.44%), aminoguanidine (+10.51%), galantamine (+9.15%), memantine (+9.14%; all *p* < 0.0001) and rivastigmine (+6.00%; *p* = 0.0008) (Figure 2). APs are key precursors of AGEs, which are formed by multistep processes such as dehydration, oxidation, and molecular rearrangement [16] (Figure 2).

Long-term exposure of proteins to reducing sugars leads to major changes in protein structure. Protein α-helix structures can be transformed into linear ones, which promotes the formation of insoluble βA aggregates [26]. These aggregates are linked to the pathomechanism of many neurodegenerative diseases, including AD, where deposited βA and AGEs increase oxidative stress, inflammation, and cell damage [51].

The βA level was lower in BSA vs. the positive control (−27.01%; *p* < 0.0001). Compared with BSA and fructose, only donepezil increased βA (+7.88%; *p* = 0.0447), whereas aminoguanidine reduced it (−17%; *p* < 0.0001); the remaining drugs showed no significant changes (*p* > 0.05) (Figure 2).

Fluorescent AGEs include ARG, formed mainly by the reaction of arginine with MGO and other reactive protein carbonyls, which are products of glucose metabolism; CRO, which is a cross-bridge between proteins; VES, formed when amino acids react with products of autooxidation; PEN, which is a marker of crosslinked proteins. Total AGEs is a term used to describe the total fluorescent AGEs in a sample [39]. AGEs can alter the structural and functional properties of proteins, leading to stiffening, reduced solubility, and loss of biological activity [11].

ARG levels did not differ between BSA and the positive control. Versus BSA samples incubated with fructose, higher levels occurred with donepezil (+198.1%), memantine (+26.31%), rivastigmine (+13.98%), and galantamine (+13.3%), while lower levels were observed with lamotrigine (−58.93%), aminoguanidine (−43.66%), and carbamazepine (−17.87%) (all *p* < 0.0001) (Figure 2). Compared with BSA incubated with fructose, higher levels were observed with donepezil for all markers except CRO (VES +466.6%, PEN +1316%, AGEs +201.8%), with galantamine for CRO and AGEs (+24.76%, +21.09%), and with memantine for all markers except AGEs (CRO +16.25% (*p* = 0.0167), VES +12.86% (*p* = 0.0085), PEN +17.21% (*p* = 0.0161), AGEs +13.96% (*p* = 0.0022)). Lower levels were observed with aminoguanidine for CRO (−55.25%), VES (−66.22%), PEN (−52.17%), and AGEs (−63.98%), and with lamotrigine for PEN (−35.26%); all *p* < 0.0001 except where noted (Figure 2).

DT is formed by the oxidative fusion of two Tyr residues under the influence of reactive oxygen species (ROS), such as the hydroxyl radical, and enzymes, such as myeloperoxidase (MPO). It indicates oxidative stress and protein damage in inflammatory and neurodegenerative diseases. KN is a product of the oxidative degradation of Trp in the kynurenine pathway, formed under the influence of enzymes such as indoleamine 2,3-dioxygenase (IDO) and tryptophan 2,3-dioxygenase (TDO). Its production increases during inflammation. It plays a key role in Trp metabolism and nervous system function. NFK is an early degradation product of Trp, formed under the influence of reactive oxygen species, ultraviolet (UV) radiation, and the enzymes IDO and TDO. It is a precursor of kynurenine and a marker of oxidative stress, involved in inflammatory processes and age-related diseases [52,53].

DT, KN, and NFK levels were consistently lower in BSA (DT −54.64%, KN −68.98%, NFK −73.89%) and aminoguanidine (DT −61.95%, KN −53.1%, NFK −55.48%) versus the positive control (all *p* < 0.0001, except DT: BSA *p* = 0.0028; aminoguanidine *p* = 0.0008). In contrast, donepezil markedly increased all markers (DT +1418%, KN +28.69%, NFK +391.9%), while galantamine increased KN and NFK (+67.74%, +94.55%) and memantine elevated KN and NFK (+21.26% and +16.14%; *p* = 0.0011 for NFK; others *p* < 0.0001). Carbamazepine also resulted in higher NFK levels (+34.32%; *p* < 0.0001) (Figure 2).

#### 3.1.2. Ribose-Induced Glycation

Ribose has a higher glycation potential compared to fructose and glucose, resulting in greater production of AGEs and higher cytotoxicity in cell models [54]. It shows selective reactivity to lysine (Lys) residues, leading to significant protein conformation changes [54].

**Figure 3:**
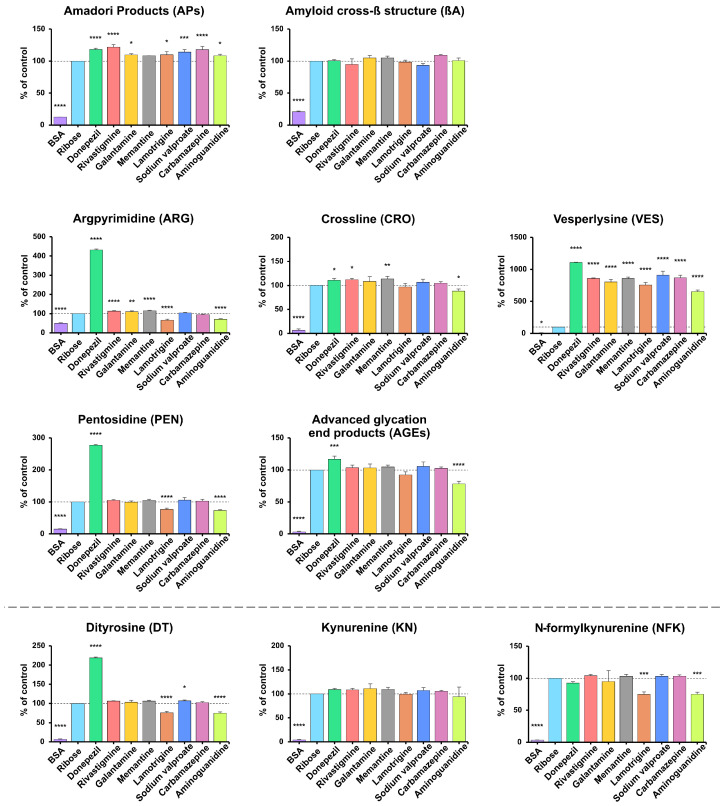
The inhibitory effects of donepezil, rivastigmine, galantamine, memantine, lamotrigine, sodium valproate, carbamazepine, and aminoguanidine on protein glycation and glycoxidation in ribose-induced glycation. APs, Amadori products; βA, amyloid cross-β structure; ARG, argpyrimidine; CRO, crossline; DT, dityrosine; KN, kynurenine; NFK, N-formylkynurenine; PEN, pentosidine; AGEs, advanced glycation end products; VES, vesperlysine; BSA, bovine serum albumin; fructose, albumin glycation induced by fructose; * *p* < 0.05 vs. positive control (glycating agent); ** *p* < 0.01 vs. positive control (glycating agent); *** *p* < 0.001 vs. positive control (glycating agent); **** *p* < 0.0001 vs. positive control (glycating agent).

AP content was significantly lower in BSA samples vs. the positive control (−87.4%; *p* < 0.0001). Compared with BSA incubated with ribose, APs levels were higher for all substances except memantine, with the highest values for rivastigmine (+21.85%), donepezil (+18.09%), and carbamazepine (+17.97%), followed by sodium valproate (+13.98%), lamotrigine (+10.03%), galantamine (+9.74%), and aminoguanidine (+8.61%) (all *p* < 0.05; most *p* < 0.0001). βA levels were significantly lower only in BSA samples (−79.2%; *p* < 0.0001) (Figure 3).

ARG, CRO, VES, PEN, and AGEs levels were significantly lower in samples containing BSA (ARG −49.97%, CRO −93.61%, VES −96.04%, PEN −84.73%, AGEs −96.78%; all *p* < 0.0001 except VES *p* = 0.0195). Versus the positive control, lower levels were also observed with lamotrigine and aminoguanidine for ARG (−33.88%, −28.85%), with aminoguanidine for CRO (−11.61%), and with aminoguanidine and lamotrigine for PEN (−26.91%, −23.5%) (all *p* < 0.05). Compared to control samples with BSA and ribose, the highest levels were observed in samples with donepezil (ARG +331.1%, CRO +10.77%, VES +1009%, PEN +177.3%, AGEs +16.58%; all *p* < 0.0001 or *p* < 0.001). Significant VES elevations were also observed with sodium valproate (+810.4%), carbamazepine (+769.9%), memantine (+760.1%), rivastigmine (+758.5%), galantamine (+702.2%), lamotrigine (+657.5%), and aminoguanidine (+553.8%) (all *p* < 0.0001). Higher ARG levels were also found in samples with memantine, rivastigmine, and galantamine for ARG (+15.47%, +13.46%, +10.36%), and with memantine and rivastigmine for CRO (+13.59%, +11.9%) (all *p* < 0.05) (Figure 3).

DT, KN, and NFK concentrations were significantly lower in samples containing BSA (DT −93.21%, KN −95.73%, NFK −96.5%; all *p* < 0.0001). Compared to control samples incubated with BSA and ribose, lower levels were also observed with aminoguanidine and lamotrigine for DT (−25.21%, −24.44%) and NFK (−24.88%, −25.39%) (all *p* < 0.001). In contrast, DT levels were higher with donepezil (+118.9% (*p* < 0.0001)) and mildly elevated with sodium valproate (+6.95% (*p* = 0.0477)) (Figure 3).

#### 3.1.3. MGO-Induced Glycation

MGO is mainly formed as a by-product of glycolysis and is a key precursor of protein glycation, leading to the formation of AGEs [55].

**Figure 4:**
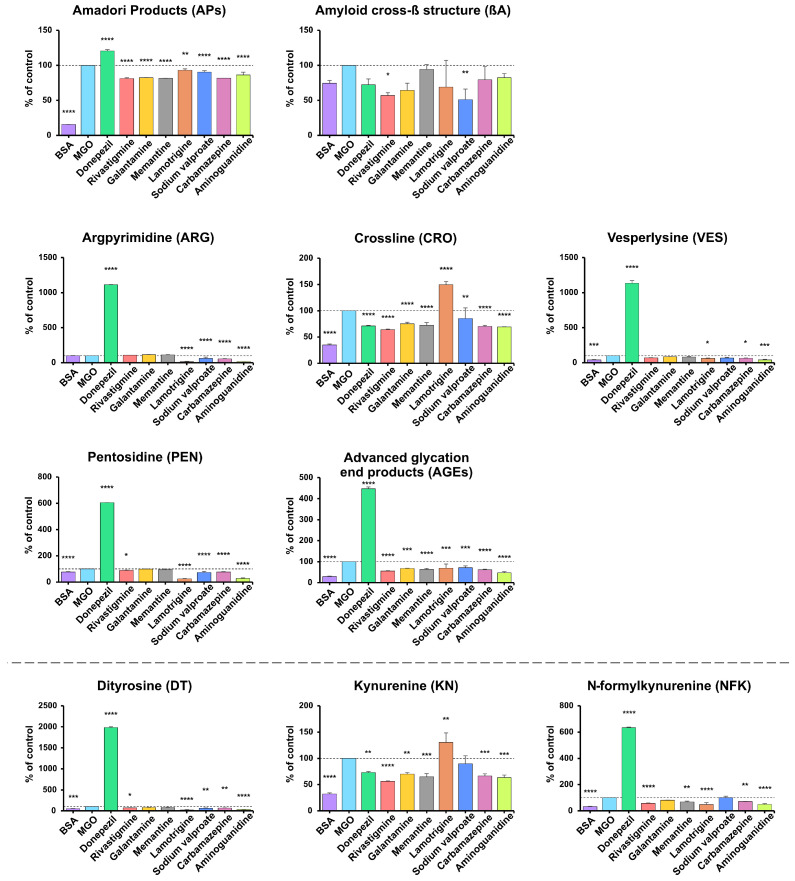
The inhibitory effects of donepezil, rivastigmine, galantamine, memantine, lamotrigine, sodium valproate, carbamazepine, and aminoguanidine on protein glycation and glycoxidation in MGO-induced glycation. APs, Amadori products; βA, amyloid cross-β structure; ARG, argpyrimidine; CRO, crossline; DT, dityrosine; KN, kynurenine; NFK, N-formylkynurenine; PEN, pentosidine; AGEs, advanced glycation end products; VES, vesperlysine; BSA, bovine serum albumin; MGO, methylglyoxal-induced glycation; * *p* < 0.05 vs. positive control (glycating agent); ** *p* < 0.01 vs. positive control (glycating agent); *** *p* < 0.001 vs. positive control (glycating agent); **** *p* < 0.0001 vs. positive control (glycating agent).

AP levels were significantly lower in BSA samples vs. the positive control (−84.32%). Compared with BSA incubated with MGO, the most significant reductions were observed with rivastigmine, memantine, carbamazepine, and galantamine (−18.73% to −17.59%), followed by aminoguanidine (−13.66%) and moderate decreases with sodium valproate and lamotrigine (−9.63%, −6.95%); all *p* < 0.0001 except lamotrigine *p* = 0.0027. In contrast, donepezil increased APs by 20.54% (*p* < 0.0001). βA content showed no significant change in BSA or aminoguanidine samples, but was lower with sodium valproate (−48.98%) and rivastigmine (−43.12%) (*p* < 0.05), while other drugs produced non-significant decreases compared with BSA + MGO (Figure 4).

Across all markers (ARG, CRO, VES, PEN, AGEs), the lowest levels were generally observed in samples with BSA and aminoguanidine (ARG −84.25%, CRO −65.18%/−30.57%, VES −60.4%/−58.32%, PEN −23.13%/−72.44%, AGEs −70.28%/−52.99%; all *p* < 0.001), while BSA showed no significant change in ARG. Lamotrigine markedly reduced ARG (−86.86%), PEN (−75.64%), AGEs (−30.18%), and VES (−38.78%), but increased CRO (+49.63%) (all *p* < 0.05). Donepezil caused the most significant increases in all biomarkers (ARG +1014%, VES +1036%, PEN +504.6%, AGEs +347.8%; all *p* < 0.0001), while decreasing CRO (−28.81%, *p* < 0.0001). Carbamazepine and sodium valproate caused moderate reductions in ARG (−44.88%, −38.41%), PEN (−22.55%, −27.05%), and AGEs (−37.92%, −28.16%) (all *p* < 0.001). Memantine and galantamine produced smaller decreases in CRO (−27.6% and −24.82%) and AGEs (−36.94% and −31.97%). Rivastigmine reduced CRO (−36.03%), PEN (−11.26%), and AGEs (−43.99%) (*p* < 0.05) (Figure 4).

DT, KN, and NFK levels showed clear marker-specific trends. DT concentrations were highest with donepezil (+1884%) and lowest with lamotrigine (−74.67%), aminoguanidine (−67.78%), BSA (−45.87%), sodium valproate (−35.18%), carbamazepine (−31.18%), and rivastigmine (−29.41%) vs. BSA + MGO (all *p* < 0.05; most *p* < 0.0001). For KN, the lowest levels occurred in BSA (−67.63%), with further reductions from rivastigmine (−43.88%), aminoguanidine (−36.72%), memantine (−34.95%), carbamazepine (−33.49%), galantamine (−29.91%), and donepezil (−26.96%) (all *p* < 0.005). In contrast, NFK was highest with donepezil (+533.8%) and lowest with BSA (−67.46%), lamotrigine (−52.01%), aminoguanidine (−49.96%), rivastigmine (−42.38%), memantine (−31.08%) and carbamazepine (−28.37%) (*p* < 0.05; most *p* < 0.0001) (Figure 4).

### 3.2. BSA Oxidation Model

In the BSA oxidation model, we investigated whether the tested drugs could modulate oxidative modifications of BSA induced by ChT. BSA + ChT served as the positive control, whereas BSA alone and NAC served as reference conditions representing basal and antioxidant-protected states, respectively. We quantified TTs as an indicator of thiol oxidation, and PCs together with AOPPs as markers of protein oxidative damage.

ChT is a potent synthetic oxidizing agent. It is the source of the main physiologically active chlorine species (ACS): hypochlorous acid (HClO) and chloramine (NH_2_Cl) [56].

**Figure 5:**
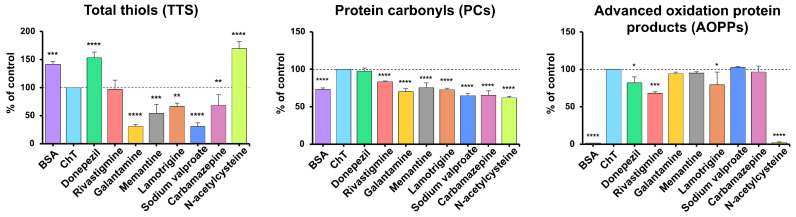
The inhibitory effects of donepezil, rivastigmine, galantamine, memantine, lamotrigine, sodium valproate, carbamazepine, and N-acetylcysteine (NAC) on protein oxidation markers in ChT-induced oxidative stress models. AOPPs, advanced oxidation protein products; BSA, bovine serum albumin; ChT, chloramine T; PCs, protein carbonyls; TTs, total thiols; * *p* < 0.05 vs. positive control (oxidizing agent); ** *p* < 0.01 vs. positive control (oxidizing agent); *** *p* < 0.001 vs. positive control (oxidizing agent); **** *p* < 0.0001 vs. positive control (oxidizing agent).

TTs were significantly higher in samples with NAC (+69.29%), donepezil (+53.33%) and BSA (+41.37%; all *p* < 0.001), while lower TTs were observed in sodium valproate (−69.27%), galantamine (−69.13%), memantine (−45.68%), lamotrigine (−33.61%) and carbamazepine (−31.34%) versus BSA + ChT (all *p* < 0.01; *p* < 0.0001 for sodium valproate and galantamine) (Figure 5). Reduced TTs, resulting from their oxidation to disulfides, are often accompanied by elevated AOPPs, indicating intensified oxidation [57].

PCs levels were significantly lower in all samples compared with (BSA + ChT), with the most significant reductions in NAC –38.1% (a well-known antioxidant), sodium valproate (−35.33%), carbamazepine (−34.51%), galantamine (−29.83%), lamotrigine (−27.34%), BSA (−26.86%), memantine (−24.34%) and rivastigmine (−16.47%) (all *p* < 0.0001), while donepezil showed no significant change (Figure 5).

AOPPs are formed by the oxidation of amino acids with free amino, amide, or hydroxyl groups, such as arginine, Lys, or Trp. AOPPs include oxidatively modified albumin, fibrinogen, and lipoprotein derivatives, as well as aggregates containing PCs, excess modified Try, arginine, Lys, and amino acids containing sulfur. AOPPs content was significantly lower in samples with BSA (−98.43%) and NAC (−97.83%), followed by moderate decreases with rivastigmine (−31.84%), lamotrigine (−20.65%), and donepezil (−17.78%) versus the positive control (*p* < 0.05; most *p* < 0.001) (Figure 5).

### 3.3. In Silico Analyses

Molecular docking is an in silico computational technique used to predict the preferred orientation of a small molecule (ligand) when it binds to a target protein and to estimate the strength of this interaction [47,58]. In the present study, docking simulations were performed as a complement to the BSA glycation model to characterize the potential binding modes and affinities of the investigated drugs (donepezil, rivastigmine, galantamine, memantine, lamotrigine, sodium valproate, and carbamazepine) toward proteins involved in carbonyl stress and glycation pathways. The targets included BSA, glycosidases such as α-amylase (αA), α-glucosidase (αG) and sucrase-isomaltase (SI), as well as AGEs-related signaling proteins: receptor (RAGE), signal transducer and activator of transcription (STAT), p38 mitogen-activated protein kinase (p38 MAPK), and nuclear factor kappa B (NF-κB).

The binding energy to BSA depends on the electrostatic energy and van der Waals forces, which are responsible for the attraction between ligand and protein, and the solvation energy, which affects the stability of the complex in aqueous media. The stability of the complex can be assessed by root mean square deviation (RMSD), where lower values indicate greater stability, and trajectory analysis, which shows whether the complex maintains stability under dynamic conditions. Key features of BSA include hydrophobic and hydrophilic regions of binding sites and potential competition of the ligand with other substances, such as glycating agents, which can affect binding efficiency. The specificity of the interaction depends on the number of hydrogen bonds and the characteristics of amino acid residues, such as Lys or Tyr, which can be crucial in inhibiting protein glycation. Further studies include interactions with other AGEs pathway proteins (NF-κB, RAGE, STAT, etc.) and glycation enzymes, such as αA and αG. Molecular dynamics (MD) simulations are essential for evaluating the stability of complexes under biological conditions [48].

#### 3.3.1. Binding Affinity of Tested Drugs and Their Antiglycative Potential

A molecular docking study analyzed interactions between selected drugs and BSA, considering the binding affinity, number of polar bonds, and amino acid residues involved. Docking simulations indicated that carbamazepine, donepezil, and galantamine had the highest binding affinity of −8.8 kcal/mol, −8.6 kcal/mol, and −8.2 kcal/mol, respectively. Rivastigmine, lamotrigine, and memantine had moderate results, with memantine forming a polar bond with Tyr-161, which may increase its effect (Table 3).

#### 3.3.2. Results of Molecular Docking Simulations Between Study Drugs and Glycosidases

Donepezil and carbamazepine had the highest binding energies with αA, of −8.5 kcal/mol and −8.7 kcal/mol, respectively; donepezil did not form potential polar bonds, which may limit the stability of the resulting complex. Carbamazepine formed two polar bonds with His-299 and Glu-233 residues, suggesting potentially better stability. Galantamine, lamotrigine, and memantine showed a moderate affinity for αA, with binding energies ranging from −6.2 to −7.1 kcal/mol. Polar bonds were present, indicating potentially fairly stable complexes. For αG, donepezil and carbamazepine again showed the highest affinity, with binding energies of −8.2 kcal/mol and −8.0 kcal/mol, respectively. Donepezil formed one polar bond with the Lys-398 residue, and carbamazepine formed one with Asp-333, suggesting good stability of the complexes. Galantamine, lamotrigine, and memantine showed moderate results, with binding energies ranging from −6.5 to −7.9 kcal/mol. The presence of polar bonds with residues, such as Asn-301, Leu-300, or Pro-602, for example, confirmed the stability of the complexes. For SI, the highest binding energies were observed for carbamazepine (−7.1 kcal/mol), donepezil (−6.5 kcal/mol), and galantamine (−6.4 kcal/mol). Carbamazepine formed two polar bonds with the Gln-48 residue, and galantamine formed two bonds with Asn-43 and Arg-282, indicating the potential stability of the mixtures. Donepezil did not form polar bonds. Lamotrigine reached –6.2 kcal/mol and formed polar bonds with Ile-70 and Gln-48 residues, suggesting moderate stability (Table 3).

#### 3.3.3. Results of Molecular Docking Simulations Between Study Drugs and Proteins of Signaling Pathways Activated by AGEs

RAGE, STAT, and NF-κB are key signaling pathway proteins associated with the action of AGEs. The results of molecular docking simulations indicate that the investigational drugs show different levels of binding affinity to these proteins. For RAGE, galantamine (−7.4 kcal/mol) achieved the highest affinity, followed by carbamazepine (−7.3 kcal/mol) and donepezil (−7.2 kcal/mol). Carbamazepine formed three polar bonds with Asn-12, Asp-14, and GLC-1 residues, indicating greater stability of the molecular complex thus formed. For STAT, carbamazepine achieved the highest binding affinity (−7.4 kcal/mol) with one polar bond (Glu-192). Donepezil (−7.0 kcal/mol) and lamotrigine (–6.2 kcal/mol) also demonstrated good stability. Lamotrigine formed five potential polar bonds (Asp-92, Gly-162, Arg-157, His-97, Glu-101), while donepezil formed three (Ser-25, Asp-24, Asn-93). For p38, donepezil, galantamine, and carbamazepine had the highest binding energies (−7.6 kcal/mol and −7.2 kcal/mol). Of the above three, galantamine is likely to form the most stable complexes due to its three potential polar bonds (Gly-85, Leu-86, His-107). Lamotrigine (−6.9 kcal/mol) also showed stability due to multiple polar bonds. For NF-κB, donepezil demonstrated the highest binding affinity (−9.5 kcal/mol) with four polar bonds (Ser-226, Lys-221, Tyr-285, DG-608), indicating a very stable molecular complex. Carbamazepine (−8.5 kcal/mol) also showed very high binding stability with two polar bonds. Galantamine (−7.8 kcal/mol), lamotrigine (−7.2 kcal/mol), rivastigmine (−6.7 kcal/mol), and memantine (−6.1 kcal/mol) demonstrated moderate stability (Table 3).

### 3.4. Systematic Review

The literature review identified 152 publications meeting the initial Medline (PubMed) search criteria. At the identification stage, 128 articles were excluded because they did not meet the inclusion criteria (e.g., no analyzed association, incorrect population, inappropriate keywords after deeper analysis). Twenty-four publications were shortlisted for further selection. At the accessibility assessment stage, one report was excluded due to limited access to the full text. Five of the 23 analyzed publications were excluded at the eligibility assessment stage, mainly due to methodological issues or failure to meet the inclusion criteria. Finally, 18 articles were included in the systematic review. The study selection scheme is presented in Figure 1.

The analyzed studies focused on the potential antiglycative properties of donepezil, rivastigmine, galantamine, memantine, lamotrigine, sodium valproate, and carbamazepine. The studies used in vitro, animal, and clinical models, analyzing drug interactions with key metabolic pathways such as RAGE and NF-κB. They assessed the effect of the drugs on AGEs levels, activity of antioxidant enzymes, reduction in oxidative stress and inflammation, and their potential role in neurodegenerative diseases, such as diabetes and metabolic complications. Biochemical methods (e.g., ELISA, Western blot) and molecular docking modeling were utilized to predict drug interactions with target proteins. Most clinical studies had Oxford Centre for Evidence-Based Medicine (CEBM) Level of Evidence 3 or 4, with only three prospective cohort studies. Thirteen studies (72.2%) rated “good”, and five (27.8%) rated “fair” in the quality assessment using the NHLBI (NIH) guidelines. The results of the analysis are presented in Table 4.

The detailed results of the literature review are presented in the Supplementary Material (Table S1).

## 4. Discussion

This study was the first to compare the antiglycative and antiglycoxidative effects of selected drugs used to treat AD and other neuropsychiatric disorders. An in vitro model of glycated and oxidized albumin was used to pursue this objective. Protein glycation was induced by agents with the highest glycation potential (fructose, ribose, and MGO) [49,54]. In silico molecular docking was also performed to predict the interactions of the drugs under investigation with key proteins, such as RAGE and mediators of the AGEs signaling pathway [18].

In terms of antiglycative activity, lamotrigine was found to be the most effective, significantly reducing the production of glycation products (ARG, PEN, VES, total AGEs) and glycoxidation products (DT, NFK, PCs, AOPPs). Lamotrigine is a voltage-gated sodium channel blocker that stabilizes neuronal membranes and modulates the release of neurotransmitters. It is used to treat epilepsy and bipolar disorder, and its biological activity is mainly due to the action of the parent drug, not the metabolites [77]. We speculate that lamotrigine may reduce the effects of protein glycation by neutralizing glycation intermediates such as MGO. It may also impede the binding of reducing sugars to proteins, which limits modifications of lysine and arginine residues, affecting their stability and protein-binding properties [78,79]. The structure of the lamotrigine molecule is based on a 1,2,4-triazine ring [80], which may be associated with its potential antioxidative and antiglycative properties. Indeed, 1,2,4-triazines have been shown to scavenge free radicals, chelate transition metal ions, and modulate antioxidative enzymes [81]. In vivo, 1,2,4-triazines increase the expression of antioxidative enzymes (heme oxygenase 1 (HO-1), glutamate–cysteine ligase (GCL), and glutathione peroxidase (GPx)) by inhibiting the proinflammatory NF-κB pathway. They also enhance the activity of nuclear factor erythroid 2-related factor 2 (Nrf2), which is crucial for defense against oxidative stress-related damage [82,83]. This study demonstrated lamotrigine has even more potent antiglycative properties than aminoguanidine in some models, especially with regard to markers such as ARG, PEN, DT, and NFK. Aminoguanidine is considered one of the most effective compounds with antiglycative activity [84] (Figure 2, Figure 3 and Figure 4). The antiglycative mechanism of aminoguanidine is based on its interaction with reactive carbonyl species (RCS), including MGO, GO, and 3-deoxyglucosone, which prevents the formation of AGEs [84]. Unfortunately, due to its high cytotoxicity, aminoguanidine is not used in vivo.

Rivastigmine, carbamazepine, and sodium valproate also showed significant, albeit weaker, antiglycative effects than lamotrigine, particularly in the MGO-induced model, where they reduced APs, total AGEs, CRO, PEN, and/or glycoxidation markers (DT, KN) (Figure 2, Figure 3 and Figure 4). However, these effects were not consistent across all conditions. In fructose- and ribose-induced glycation, their impact on early (APs) and final (total AGEs) glycation products was limited or absent, indicating only weak to moderate antiglycative and antiglycoxidative activity. At the same time, early glycation products remain unchanged or even increase, reflecting stage-specific modulation of the Maillard reaction and glycoxidation cascade. Galantamine is a good example of such context-dependent behavior: in the MGO-based model, it reduced APs, total AGEs, CRO and KN levels, whereas in fructose- and ribose-induced glycation it predominantly showed proglycative effects, increasing several AGEs (e.g., APs, ARG) and glycoxidation markers (KN, NFK) (Figure 2, Figure 3 and Figure 4). This fact is not surprising because individual sugars and aldehydes have different glycative potential, and biomarkers of glycation and glycoxidation reflect various stages of the Maillard reaction. Therefore, to avoid incorrect conclusions, it is essential to evaluate the antiglycative properties of new substances using multiple models and biomarkers [32,33,36].

Conversely, donepezil markedly intensified glycation and glycoxidation in each model. Indeed, we observed significantly higher levels of carbonyl (ARG, PEN, VES, DT, NFK) and oxidative stress markers (PCs, AOPPs) in donepezil-treated BSA samples, indicating a pronounced proglycative and pro-oxidative profile. Donepezil is an AChEI used to treat mild to moderate dementia [85]. It contains N-benzylpiperidine and indan-2-one [85,86], which can interact with RCS, potentially enhancing the formation of glycation products. In more detail, these structural motifs may interact with aromatic and basic amino acid residues, promoting covalent adduct formation and halogen/π–π interactions that stabilize protein complexes through electrostatic and polarizing interactions with protein carbonyls [87,88]. These bonds can promote protein conformation in a way that facilitates the exposure of RCS to glycating agents, potentially increasing susceptibility to glycation modifications [89]. Increased levels of key glycation and glycoxidation markers, such as APs, βA, ARG, VES, DT, and PEN, suggest that donepezil may induce carbonyl stress in vitro (Figure 2, Figure 3 and Figure 4). Other AChEIs, such as galantamine and rivastigmine, also had a proglycative effect. However, it was not as strong as that of donepezil and was not observed in all models. Importantly, our docking analysis is consistent with these findings, as donepezil showed high binding affinity for BSA and for AGEs-related targets, particularly NF-κB and RAGE (Table 3). This suggests that donepezil can stabilize ligand–protein complexes involved in proinflammatory and pro-oxidative signaling, which may further contribute to the increase in glycation and glycoxidation markers observed in our BSA model. Interestingly, the body can trigger an adaptive response in vivo that limits drug-induced carbonyl stress. The Nrf2 pathway plays a key role in protecting against carbonyl stress. In response to oxidative stress and reactive aldehydes (e.g., MGO), it induces the expression of detoxification enzymes such as HO-1, NAD(P)H: quinone oxidoreductase-1 (NQO-1), and glutathione S-transferase (GST), strengthening antioxidant defense [90]. At the same time, the activity of glyoxalase 1 and 2 (Glo1, Glo2) increases, which catalyze the neutralization of MGO to the less reactive D-lactate, limiting its participation in the formation of AGEs [91]. In addition, RAGEs are downregulated, resulting in inhibition of NF-κB and oxidative stress [92]. Therefore, evaluating the antiglycative effect of donepezil in a clinical setting is essential.

The BSA oxidation model with ChT enables the controlled modeling of oxidative modifications of proteins, such as oxidation of amino acid residues, formation of disulfide bonds, and changes in the spatial structure of the protein (secondary and tertiary) [56,93]. This study demonstrated that lamotrigine significantly reduced the production of PCs and AOPPs but was less effective than NAC (Figure 5). NAC is a strong antioxidant that reacts with oxidants (e.g., hypochlorous acid and hydroxyl radicals) to form protective adducts that prevent further oxidation of proteins. That compound removes free oxygen radicals and exhibits binding properties to pro-oxidative metal ions [94]. In this study, rivastigmine had similar results to lamotrigine. In turn, donepezil significantly increased the concentrations of both PCs and AOPPs (Figure 5). Although further studies are necessary, the antiglycative activity of the studied anti-dementia drugs may be due to their antioxidative effects.

The experimental conditions used in our glycation and oxidation models involved supraphysiological concentrations of glycating and oxidizing agents (0.5 M fructose or ribose, 2.5 mM methylglyoxal, and 20 mM chloramine T), together with a fixed concentration of the tested compounds and reference inhibitors (1 mM). Such concentrations are widely employed in in vitro glycation assays to accelerate the formation of early glycation products and AGEs within a feasible incubation time and to increase the analytical robustness of the measurements [32,33,36]. Therefore, the present experiments are best interpreted as a comparative screening of the relative antiglycative and antioxidative potential of the tested drugs under conditions of pronounced glyco-oxidative stress, rather than as a direct reflection of in vivo pharmacokinetics or clinical exposure. In physiological settings, where sugar and reactive carbonyls are markedly lower, and additional factors such as protein turnover, tissue distribution, metabolism, and blood–brain barrier penetration modulate drug action, the magnitude and possibly the direction of antiglycative or proglycative effects may differ [59,60,64,79]. This limitation should be taken into account when extrapolating our findings to neurodegenerative diseases and highlights the need for future studies using lower, more physiological concentrations and relevant neuronal and glial cell models to verify whether the drug-specific profiles observed here are reproduced in a cellular context and in vivo.

Although BSA is a widely used and convenient in vitro model of protein glycation, it only partially reflects glycation processes relevant to neurodegeneration. BSA differs substantially from neuronal proteins such as Tau, α-synuclein, and Aβ, which are intrinsically disordered and aggregation-prone, and whose glycation critically affects oligomerization and toxicity. Future studies should employ Tau-, α-synuclein-, or Aβ-based models to reflect neurodegenerative mechanisms better.

A molecular docking analysis is an in silico method used to predict the interaction of a molecule (e.g., a drug) with a target protein. It is widely conducted to design new drugs and predict their biological activity [48]. In this study, donepezil, carbamazepine, and galantamine showed the highest affinity for BSA. Donepezil showed particular activity due to the formation of numerous polar bonds. Considering the glycosidases involved in the digestion of carbohydrates in the gastrointestinal tract, donepezil and carbamazepine achieved the highest binding energies, and the stability of their complexes was enhanced by specific amino acid interactions. The tested substances also interacted with key proteins of the AGEs pathway. Donepezil had the highest affinity for NF-κB. Carbamazepine and galantamine showed a high affinity for RAGE, possibly contributing to its activation. Due to the numerous polar bonds with STAT, lamotrigine can stabilize that complex and influence the regulation of inflammatory processes. Indeed, the RAGE, STAT, NF-κB, and p38 signaling pathways play a key role in the pathogenesis of neurodegenerative diseases, mainly by stimulating inflammation and oxidative stress. The interaction of AGEs with RAGE activates a signaling cascade, leading to increased neuroinflammation [19]. Modifying these mechanisms, especially by inhibiting RAGE, may be a promising therapeutic strategy to limit inflammatory processes and slow down the progression of neurodegenerative diseases [20,27]. Although a molecular docking analysis may suggest potential agonistic or antagonistic properties of the substances under study, it does not allow a clear determination of their actual in vivo effect. Therefore, a further experimental analysis of the drug effect is necessary [95] (Table 2 and Table 3).

The systematic review of the literature conducted as part of this study indicated that out of the tested AChEIs, rivastigmine [69,70] and galantamine [63,64] showed the greatest efficacy in reducing AGEs levels [63,64,69,70], compared to donepezil [60,61,69]. In clinical trials, AD patients treated with rivastigmine had significantly lower serum AGEs levels than other treatment groups [69,70], indicating its potential antiglycative effect. That mechanism may be related to the modulation of the cholinergic system and reduced activation of the RAGE pathway, which limits chronic inflammation and carbonyl stress [69,70]. In turn, galantamine, in addition to inhibiting AChEIs, reduced the levels of glycation biomarkers, such as carboxymethyllysine (CML) and carboxymethylarginine (CMA), especially in animal models with chronic cerebral ischemia [64]. In 2018, Wazea et al. showed that galantamine reduced RAGE expression and limited NF-κB activation, indicating its protective properties against glycation-induced neurodegeneration [63] (Table 4).

During the literature review, the most diverse results were obtained for donepezil. In the animal models, that drug significantly reduced the level of AGEs in the hippocampus and cerebral cortex [60], reduced Aβ1–42 levels [61,62] and RAGE expression, and showed neuroprotective properties [62]. In turn, the use of donepezil in the animal model of STZ-induced diabetes resulted in higher levels of HbA1c and Aβ1–42 in the blood, suggesting the intensification of glycation under hyperglycemia conditions [61]. This is consistent with the findings of this study, which confirms the proglycative effect of the drug when using reducing sugars and BSA as a model protein. Meanwhile, memantine, an NMDA receptor antagonist, dose-dependently inhibited the degradation of collagen II and aggrecan induced by AGEs [59] (Table 4). Overall, when juxtaposed with our BSA-based findings, the systematic review suggests a broadly consistent pattern: rivastigmine and galantamine show a relatively favorable antiglycative profile, whereas the effects of donepezil are context-dependent and may become proglycative under conditions of pronounced carbonyl stress or hyperglycemia. In contrast, our data indicate strong antiglycative and antioxidative effects of lamotrigine, despite the scarcity of prior reports, whereas memantine, carbamazepine, and sodium valproate display only modest or model-dependent activity. This integrated view highlights both areas of agreement between experimental and clinical/animal data and critical gaps that warrant further in vivo and clinical investigation.

Finally, the limitations of the study should be discussed. Despite its structural similarity to human albumin, BSA does not mimic the complex matrix of blood serum, making it difficult to translate the results to clinical settings [18,96]. The antiglycative effect may depend on the concentration of the tested drugs. While some substances retain their efficacy in a wide range of concentrations, others show optimal activity within a well-defined concentration range. This means the need to precisely select the concentration of each substance to maximize its beneficial effects while minimizing the risk of adverse events [97,98]. This study selected incubation conditions and reagent concentrations based on previous kinetic analyses to match high levels of glycating agents [99,100]. The use of the in vitro model enables rapid modeling of biochemical processes, although it limits the translation of results into physiological conditions. The lack of analyses of toxicity and long-term side effects of the drugs under study, as well as the narrow scope of molecular analysis, further complicate the practical application of the results. Therefore, subsequent studies are necessary using both in vitro and in vivo models. Our work evaluates many anti-dementia drugs and identifies those that are of great practical importance for further research.

## 5. Conclusions

This study indicates a weak/moderate antiglycative effect of anti-dementia drugs. Lamotrigine proved to be the most effective among the examined substances. This drug reduced concentrations of selected biomarkers of glycation and glycoxidation, particularly in the MGO-induced glycation model. On the other hand, donepezil significantly increased protein glycoxidation in each model analyzed. Further research should include in vivo experiments and clinical studies incorporating molecular analyses of signaling proteins associated with neurodegeneration. It will also be important to investigate the synergistic effects of anti-dementia drugs and their effectiveness in different patient groups, such as patients with neurodegenerative diseases and comorbid diabetes.

## Figures and Tables

**Table 1:** Basic chemical information of the study compounds. The table presents the chemical names, CAS numbers, and chemical formulas of study compounds, including acetylcholinesterase inhibitors (donepezil, rivastigmine, galantamine), an NMDA receptor antagonist (memantine), and antiepileptic drugs (lamotrigine, carbamazepine, sodium valproate).

Drug	CAS Number	Full Chemical Name	Chemical Formula
Donepezil	120014-06-4	2-[(1-benzylpiperidin-4-yl)methyl]-5,6-dimethoxy-2,3-dihydroinden-1-one	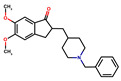
Rivastigmine	123441-03-2	[3-[(1S)-1-(dimethylamino)ethyl]phenyl] N-ethyl-N-methylcarbamate	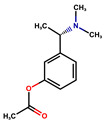
Galantamine	357-70-0	(1S,12S,14R)-9-methoxy-4-methyl-11-oxa-4-azatetracyclo [8.6.1.01,12.06,17]heptadeca-6(17),7,9,15-tetraen-14-ol	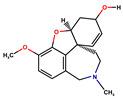
Memantine	19982-08-2	3,5-Dimethyladamantan-1-amine	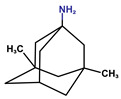
Lamotrigine	84057-84-1	6-(2,3-Dichlorophenyl)-1,2,4-triazine-3,5-diamine	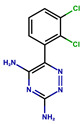
Sodium valproate	1069-66-5	Sodium 2-propylpentanoate	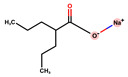
Carbamazepine	298-46-4	benzo[b][1]benzazepine-11-carboxamide	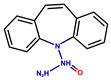

**Table 2:** Eligibility criteria for the analyzed publications.

Inclusion Criteria	Exclusion Criteria
Articles in English	Articles in languages other than English
Human, animal, and in vitro experiments	Review articles, questionnaires, and case reports
Publications on the antiglycoxidative effects of the drugs under investigation	Publications that do not address the antiglycoxidative effects of the drugs under investigation

**Table 3:** Molecular docking results of donepezil, rivastigmine, galantamine, memantine, lamotrigine, sodium valproate, and carbamazepine with target proteins involved in carbonyl stress and inflammation pathways. The table presents molecular docking results for acetylcholinesterase inhibitors (donepezil, rivastigmine, galantamine), an NMDA receptor antagonist (memantine), and antiepileptic drugs (lamotrigine, carbamazepine, sodium valproate) against key proteins implicated in neurodegenerative and inflammatory processes. The target proteins include bovine serum albumin (BSA, ID: 3V03), α-amylase (1HNY), α-glucosidase (3WY1), sucrase-isomaltase (3LPO), receptor for advanced glycation end products (RAGE, 3O3U), signal transducer and activator of transcription (STAT, 3WWT), p38 mitogen-activated protein kinase (p38 MAPK, 5OMG), and nuclear factor kappa B (NF-κB, 1A3Q). The table provides binding affinity (expressed in kilocalories per mole [kcal/mol]), the number of polar contacts, and the amino acid residues involved in ligand–protein interactions. Higher absolute values of binding affinity indicate stronger ligand–protein binding. Arg, arginine; Asn, asparagine; Asp, aspartic acid; DA, deoxyadenosine; DG, deoxyguanosine; Gln, glutamine; Glu, glutamic acid; GLC-1, glucose-1 attachment site; Gly, glycine; His, histidine; Ile, isoleucine; Lys, lysine; Pro, proline; Ser, serine; Thr, threonine; Trp, tryptophan; Tyr, tyrosine; Val, valine.

Protein	RCSB ID	Drug	Affinity (kcal/mol)	Number of Polar Contacts	Amino Acid Residues
BSA	3V03	Donepezil	−8.6	1	Arg-117
		Rivastigmine	−6.7	0	-
		Galantamine	−8.2	0	-
		Memantine	−6.3	1	Tyr-161
		Lamotrigine	−6.6	0	-
		Sodium valproate	−4.9	4	Val-120.3 × Asp-121
		Carbamazepine	−8.8	0	-
α-amylase	1HNY	Donepezil	−8.5	0	-
		Rivastigmine	−6	1	Gln-63
		Galantamine	−7.1	1	His-305
		Memantine	−6.2	2	Asp-197, Glu-233
		Lamotrigine	−6.7	2	Asp-197, Glu-233
		Sodium valproate	−4.6	3	Asp-197, His-299, Glu-233
		Carbamazepine	−8.7	2	His-299, Glu-233
α-glucosidase	3WY1	Donepezil	−8.2	1	Lys-398
		Rivastigmine	−5.6	0	-
		Galantamine	−7.9	1	Asn-301
		Memantine	−6.5	1	Leu-300
		Lamotrigine	−6.6	3	Gly-228, Leu-227, Pro-602
		Sodium valproate	−4.7	1	Asn-301
		Carbamazepine	−8	1	ASP-333
Sucrase-isomaltase	3LPO	Donepezil	−6.5	0	-
		Rivastigmine	−5	1	Thr-224
		Galantamine	−6.4	2	Asn-43, Arg-282
		Memantine	−5.2	0	-
		Lamotrigine	−6.2	2	Ile-70, Gln-48
		Sodium valproate	−5.5	3	His-213, Glu-588, Lys-214
		Donepezil	−6.5	0	-
RAGE	3O3U	Donepezil	−7.2	1	Asn-12
		Rivastigmine	−5.6	1	GLC-1
		Galantamine	−7.4	1	Asp-14
		Memantine	−6.1	1	Asp-14
		Lamotrigine	−6.1	1	Asn-12
		Sodium valproate	−4.2	2	GLC-1, Asn-12
		Carbamazepine	−7.3	3	Asn-12, Asp-14, GLC-1
STAT	3WWT	Donepezil	−7	3	Ser-25, Asp-24, Asn-93
		Rivastigmine	−5	1	Gly-162
		Galantamine	-	0	-
		Memantine	−5.9	0	-
		Lamotrigine	−6.2	5	Asp-92, Gly-162, Arg-157, His-97, Glu-101
		Sodium valproate	−4.3	2	Gln-188, Tyr-174
		Carbamazepine	−7.4	1	Glu-192
p38	5OMG	Donepezil	−7.6	1	Arg-149
		Rivastigmine	−5.9	1	Asn-201
		Galantamine	−7.2	3	Gly-85, Leu-86, His-107
		Memantine	−5.9	1	His-80
		Lamotrigine	−6.9	5	4x Gln-202, Asp-145
		Sodium valproate	−4.6	2	Val-83, His-80
		Carbamazepine	−7.2	1	Arg-149
NF-κB	1A3Q	Donepezil	−9.5	4	Ser-226, Lys-221, Tyr-285, DG-608
		Rivastigmine	−6.7	5	Lys-252, DA-607, DA-608, DA-609, Lys-221
		Galantamine	−7.8	4	3x DG-607, DG-606
		Memantine	−6.1	1	Gln-284
		Lamotrigine	−7.2	2	Gln-284, DG-608
		Sodium valproate	−4.9	4	Lys-252, 2x DA-609, DA-510
		Carbamazepine	−8.5	2	Tyr-285, Lys-252

**Table 4:** Results of a systematic review of the antiglycative properties of donepezil, rivastigmine, galantamine, memantine, lamotrigine, sodium valproate, and carbamazepine. ↑ increase; ↓ decrease..

Study Design (Model)	End-Points (Main Findings)	
**In vitro model**		
BSA–MGO–AGEs (model of AGEs-induced degradation of collagen II and aggrecan); SW1353 chondrosarcoma cells lines; memantine 5–10 µM (24 h) + AGEs 100 µg/mL (48 h).	Memantine ↑ protection against AGEs-induced degradation of collagen II and aggrecan; ↓ MMP-13, ↓ ADAMTS-4, ↓ JAK2/STAT1/IRF-1 activation; all *p* < 0.01; effect dose-dependent.	[59]
**Animal model**		
Male C57BL/6J mice; high-fat diet (HFD) 16 weeks → donepezil 3 mg/kg i.p. for 4 weeks; groups: CD, HFD, HFDD, CDD; AGEs measured in hippocampus & cortex (ELISA).	HFD ↑ AGEs vs. CD; donepezil ↓ AGEs in HFD mice (*p* < 0.01); no effect of donepezil in CD.	[60]
Male Wistar rats; STZ-induced hyperglycemia (42 mg/kg (i.p.)); groups: negative control, STZ, STZ + donepezil 10 mg/kg (i.p.); treatment from day 61; assessed blood & brain glycation biomarkers (Aβ42, β-secretase, HbA1c).	STZ: ↑ HbA1c, ↑ blood Aβ42 (*p* < 0.05), ↑ brain β-secretase (*p* < 0.05) vs. control. Donepezil: ↓ brain β-secretase (*p* < 0.05 vs. STZ), ↓ Aβ42 in brain (*p* < 0.05); no effect on blood glucose (*p* = 0.07). Blood Aβ42 remained ↑ in STZ and donepezil (*p* < 0.05); HbA1c elevated in STZ and donepezil (no reduction).	[61]
Sprague–Dawley rats; intracerebral administration Aβ1–42 (CA1) AD model; treatment for 30 days: donepezil 2 mg/kg vs. water; assessed Aβ1–42, LRP-1, RAGE (ELISA, WB, RT-qPCR, IHC).	Donepezil: ↓ Aβ1–42 in hippocampus (2.36 ± 0.28 vs. 4.17 ± 0.35 ng/mg; *p* < 0.01); ↓ RAGE expression (WB −27%; *p* < 0.05); ↓ RAGE staining (0.52 ± 0.08 vs. 0.71 ± 0.12; *p* < 0.05); strong neuroprotection.	[62]
Wistar rats; TNBS-induced colitis; groups: control, TNBS, sulfasalazine, galantamine 10 mg/kg p.o., MLA 5.6 mg/kg, MLA + galantamine; drugs for 11 days; colitis induced on day 8.	TNBS: ↑ RAGE (>2-fold; *p* < 0.001). Galantamine: ↓ RAGE by 48% vs. TNBS (*p* < 0.001), likely via α7 nAChR → ↓ NF-κB; ↓ HMGB1; anti-inflammatory effect.	[63]
APP23 transgenic mice (AD model); carotid artery stenosis–induced chronic cerebral ischemia (CCH); groups: control, APP23, APP23 + CCH, APP23 + CCH + galantamine 5 mg/kg p.o.; treatment from day 15 for 1.5–8 months; AGEs biomarkers: CML, CMA measured at 6 and 12 months.	APP23 + CCH: ↑ AGEs at 12 months (↑ CML in neurons, ↑ CMA in vessels/plaques; *p* < 0.01). Galantamine: ↓ CML by ~30% and ↓ CMA by ~25% vs. untreated APP23 + CCH (*p* < 0.05); stronger effect with longer treatment (7.5 vs. 1.5 months).	[64]
Wistar rats; 4 groups: control, L. nobilis extract 150 mg/kg bw, sodium valproate 500 mg/kg bw, valproate + *L. nobilis*; 30-day i.p. treatment; assessed glucose, HbA1c, ALT, AST, creatinine, urea; liver/kidney histology.	Valproate: ↑ glucose (116.0 ± 2.64 vs. 104.0 ± 6.0 mg/dL) and ↑ HbA1c (7.10 ± 0.10 vs. 6.73 ± 0.21 mg/dL; *p* < 0.05). *L. nobilis* extract: ↓ these valproate-induced increases (glucose, HbA1c), indicating protective antiglycative effect.	[65]
Sprague–Dawley rats; STZ-induced type 1 diabetes (single injection of STZ (75 mg/kg, i.p.)); 5 groups: control, valproate 300 mg/kg/day, STZ, STZ + valproate 150 mg/kg/day, STZ + valproate 300 mg/kg/day; 3-week p.o. treatment; outcomes: plasma glucose, insulin, HbA1c, glucose tolerance (AUC), pancreatic islet morphology, β-cell apoptosis (TUNEL), H3 acetylation; (ELISA, IHC).	STZ: ↑ glucose (620.0 ± 20.5 vs. 116.0 ± 4.0 mg/dL; *p* < 0.001) and ↑ HbA1c (7.92 ± 0.70 vs. 4.48 ± 0.25%; *p* < 0.001). Valproate 300 mg/kg: ↓ glucose (455.7 ± 69.9 mg/dL; *p* < 0.01 vs. STZ) and ↓ HbA1c to 6.88 ± 0.23 (NS). Valproate: protected β-cells (↓ apoptosis) and ↑ H3 acetylation (epigenetic improvement).	[66]
Rats; kainic acid (KA, 12 mg/kg i.p.) epilepsy model; 4 groups: PBS, KA, KA + *Urtica urens* extract (UR, 1 g/kg/p.o., 5×/week, 6 weeks), KA + valproate (250 mg/kg/p.o., 5×/week, 6 weeks); assessed hippocampal (CA1) glycation & inflammation markers (S100B, RAGE, mGluR3, MCP-1, CCR-2), histology, WB, EEG.	KA: ↑ S100B and ↑ RAGE (260.67 ± 34.89 cells/field). Valproate: ↓ S100B (112.33 ± 20.92; *p* < 0.05 vs. KA) and ↓ RAGE (102.5 ± 32.25; *p* < 0.05 vs. KA), similar to UR extract (129.17 ± 19.38). Both valproate and UR: ↓ inflammation and glycation markers in the epilepsy model.	[67]
BALB/c mice; carbamazepine p.o.: 400 mg/kg (4 days) → 800 mg/kg (day 5); assessed ALT, AST, GSH, protein carbonyls, TLR4, RAGE, IL-6/17/23; evaluated TLR4/RAGE blockade and PGE1 for hepatoprotection.	Carbamazepine: ↑ ALT/AST (*p* < 0.05), ↓ GSH (1.5 to 24 h; *p* < 0.05), ↑ protein carbonyls (*p* < 0.05), ↑ TLR4 (6 h; *p* < 0.05), ↑ RAGE (12 h; *p* < 0.05). Administration of PGE1: ↓ ALT, AST, IL-6, IL-23, IL-17 (*p* < 0.05), indicating hepatoprotection.	[68]
**Human model**		
AD patients (n = 21) vs. matched controls (n = 10); ≥24-month oral treatment: donepezil 10 mg/day, rivastigmine 9.5 mg/day, or donepezil + memantine; assessed plasma AGEs (spectrofluorimetry), endothelial dysfunction & inflammation markers.	AD: ↑ AGEs by 33% vs. controls. Rivastigmine: ↓ AGEs vs. other treatments (*p* < 0.05), though still > control. Other regimens: AGEs ↑ vs. control and not significantly different between treatments.	[69]
AD patients with comorbid diabetes vs. healthy age-matched controls; rivastigmine transdermal patches (4.6 mg daily, 6 weeks → 9.5 mg daily, 30 weeks); measured serum AGEs (ELISA/HPLC) and HbA1c.	AD: ↑ AGEs by +32% vs. controls (*p* < 0.05). Rivastigmine: ↓ AGEs by −25% after 30 weeks (*p* = 0.01). AD: ↑ HbA1c (6.7 ± 0.5% vs. 5.7 ± 0.3%; *p* = 0.03); rivastigmine: ↓ HbA1c by −15% (*p* < 0.05).	[70]
Obese adults (n = 40; BMI 30–39.9); randomized, double-blind, placebo-controlled; lamotrigine 200 mg/day p.o. vs. placebo for 26 weeks; assessed body weight, BMI, body fat, lipid profile, HbA1c, IWQOL.	Lamotrigine: ↓ body weight (*p* = 0.0623) and ↓ BMI (*p* = 0.0421); no changes in body fat or lipids. HbA1c: no effect (lamotrigine +0.1 ± 0.3% vs. placebo +0.0 ± 0.3%; *p* = 0.9093).	[71]
Cross-sectional study: patients with idiopathic generalized tonic–clonic epilepsy treated ≥1 year with valproate (400–1750 mg/day/p.o.; n = 22) or lamotrigine (100–500 mg/day/p.o.; n = 22), newly diagnosed/untreated (n = 22), and healthy controls (n = 22). Measured fasting glucose, insulin, HbA1c, HOMA-IR, lipid profile, asprosin (ELISA).	Valproate: ↑ fasting glucose (127.8 ± 11.6 vs. 91.4 ± 8.9 mg/dL; *p* < 0.01), ↑ HbA1c (6.2 ± 0.4% vs. 5.5 ± 0.3%; *p* < 0.01), ↑ insulin (12.8 ± 3.6 vs. 8.3 ± 2.4 μIU/mL; *p* < 0.05). Lamotrigine: glucose, HbA1c, asprosin comparable to controls. Valproate associated with ↑ glycation risk; lamotrigine not.	[72]
Patients (n = 125) with drug-resistant epilepsy (aged 23–85); long-term p.o. AED therapy: valproate 1000–2000 mg/day (n = 50), lamotrigine 100–400 mg/day (n = 48); oxcarbazepine 600–2400 mg/day (n = 46), carbamazepine 400–1600 mg/day (n = 37), phenytoin 200–400 mg/day (n = 32), levetiracetam 500–3000 mg/day (n = 31).Assessed CRP, HbA1c, eGFR (multivariate regression).	Lamotrigine: ↓ HbA1c by 2% (NS; *p* = 0.11), ↓ eGFR by 13% (*p* = 0.001). Valproate: ↓ CRP by 55% (*p* = 0.001), ↑ eGFR by 10% (*p* = 0.018), no HbA1c effect. Carbamazepine: no HbA1c effect (*p* = 0.24). Phenytoin: ↓ HbA1c by 4% (*p* = 0.004).	[73]
66 patients (age 4–83) with epilepsy/neuralgia on long-term oral carbamazepine; assessed carbamazepine free fraction (CBZ-FF%), glycated vs. non-glycated albumin (NGA), and age-related biochemical parameters.	With age: ↑ glycated albumin (r = 0.666; *p* < 0.001) and ↓ NGA (r = −0.459; *p* < 0.001) → ↑ CBZ-FF% (r = 0.992; *p* < 0.001). Elderly: highest glycated albumin (17.3%) + lowest NGA (3.3 g/dL) → highest CBZ-FF% (31.1%; *p* < 0.001). Carbamazepine does not alter albumin glycation.	[74]
Insulin-dependent diabetes (IDD) patients (n = 62) vs. healthy controls (n = 22); measured serum albumin (g/L), HbA1c, and valproic acid binding (100 mg/L) by equilibrium dialysis + fluorescence anisotropy.	Diabetic group: albumin 38.7 ± 1.9 g/L; HbA1c 4.1 ± 2.1%. Controls: albumin 38.6 ± 2.2 g/L; HbA1c 1.1 ± 0.5%. Valproate binding ↓ in diabetics (75.2 ± 6.7%) vs. controls (80.7 ± 4.9%; *p* < 0.001). No correlation between HbA1c and valproate binding → decrease linked to diabetes-specific albumin modification, independent of glycation.	[75]
Epilepsy patients (n = 23, both sexes, middle-aged) on long-term carbamazepine p.o. (>2 years; therapeutic serum levels) vs. healthy controls (aged 17–35); assessed hemoglobin fractions incl. Hb ASSG (glutathione–hemoglobin adduct) via ion-exchange chromatography and electrophoresis.	Carbamazepine: ↑ Hb ASSG to 2–7% (not detected in controls; *p* < 0.01). ↑ MetHb (8.5% ± 1.5% vs. 3% ± 0.8%; *p* < 0.001). ↓ thiols (SH) in Hb ASSG (0–1 SH vs. 2 SH in controls; *p* < 0.05). Strong correlation: Hb ASSG ↔ MetHb (r = 0.8; *p* < 0.01) → oxidative stress–driven ↑ glycation in long-term carbamazepine therapy.	[76]

**Abbreviations used in the table: Aβ42**, Amyloid β 42; **AD**, Alzheimer’s disease; **ADAMTS-4**, ADAM metalloproteinase with thrombospondin motifs 4; **AGEs**, advanced glycation end-products; **ALT**, alanine aminotransferase; **AST**, aspartate aminotransferase; **AUC**, glucose tolerance (area under the curve); **BSA**, bovine serum albumin; **CA1**, hippocampal region CA1; **CBZ-FF%**, carbamazepine free fraction; **CCH**, chronic cerebral hypoperfusion; **CCR2**, chemokine receptor 2; **CD**, control diet; **CMA**, carboxymethylarginine; **CML**, carboxymethyllysine; **CRP**, C-reactive protein; **EEG**, electroencephalographic recording; **eGFR**, estimated glomerular filtration rate; **ELISA**, enzyme-linked immunosorbent assay; **GSH**, glutathione; **HbA1c**, glycated hemoglobin; **Hb ASSG**, glutathione–hemoglobin adduct; **HFD**, high-fat diet; **HMGB1**, high-mobility group box 1; **HOMA-IR**, homeostatic model assessment of insulin resistance; **HPLC**, high-performance liquid chromatography; **IHC**, immunohistochemistry; **IDD**, insulin-dependent diabetes; **IL-6**, interleukin 6; **IL-23**, interleukin 23; **IL-17**, interleukin 17; **IWQOL**, Impact of Weight on Quality of Life; **JAK2/STAT1/IRF-1**, Janus kinase 2/signal transducer and activator of transcription 1/interferon regulatory factor-1; **KA**, kainic acid; **LRP-1**, lipoprotein-related receptor 1; **MCP-1**, monocyte chemoattractant protein 1; **MetHb**, methemoglobin; **MGO**, methylglyoxal; **mGluR3**, metabotropic glutamate receptor 3; **MLA**, methyl-lycaconitine; **MMP-13**, matrix metalloproteinase 13; **NGA**, non-glycated albumin; **NF-κB**, nuclear factor kappa B; **ns**, not significant; **PBS**, phosphate-buffered saline; **PGE1**, prostaglandin E1; **RAGE**, receptor for advanced glycation end-products; **RT-qPCR**, reverse transcription quantitative polymerase chain reaction; **S100B**, calcium-binding protein B; **SH**, thiols; **STZ**, streptozotocin; **SW1353**, chondrosarcoma cell line; **TLR4**, Toll-Like Receptor 4; **TNBS**, 2,4,6-trinitrobenzene sulfonic acid; **UR**, *Uncaria rhynchophylla* extract; **WB**, Western blot.

## Data Availability

The data that support the findings of this study are available from the corresponding author upon reasonable request.

## Supplementary Materials

The following supporting information can be downloaded at https://www.mdpi.com/article/10.3390/antiox14121509/s1. Table S1. Complete results of the systematic review on the antiglycative properties of donepezil, rivastigmine, galantamine, memantine, lamotrigine, sodium valproate, and carbamazepine.

## Abbreviations

The following abbreviations are used in this manuscript:

**2,4-DNPH**	2,4-dinitrophenylhydrazine
**Aβ1–42**	amyloid beta 1–42
**ACS**	active chlorine species
**AD**	Alzheimer’s disease
**ADAMTS-4**	disintegrin and metalloproteinase with thrombospondin motifs 4
**AGEs**	advanced glycation end products
**ALT**	alanine aminotransferase
**ALS**	amyotrophic lateral sclerosis
**AOPPs**	advanced oxidation protein products
**APP23**	transgenic mouse model with APP mutation associated with Alzheimer’s disease
**APs**	Amadori products
**Arg**	arginine
**ARG**	argpyrimidine
**Asn**	asparagine
**Asp**	aspartic acid
**AST**	aspartate aminotransferase
**AUC**	area under the curve
**BALB/c**	laboratory-bred mouse strain
**BMI**	body mass index
**BSA**	bovine serum albumin
**βA**	amyloid cross-β structure
**C57BL/6J**	laboratory-bred mouse strain
**CA1**	cornu ammonis region 1
**CCR-2**	C-C chemokine receptor 2
**CD**	Crohn’s Disease
**CDD**	control diet
**ChT**	chloramine T
**CMA**	carboxymethylarginine
**CML**	carboxymethyllysine
**CRP**	C-reactive protein
**CRO**	crossline
**DA-607**	deoxyadenosine
**DG**	deoxyguanosine
**DT**	dityrosine
**EEG**	electroencephalography
**eGFR**	estimated glomerular filtration rate
**ELISA**	enzyme-linked immunosorbent assay
**Gln**	glutamine
**Glu**	glutamic acid
**GLC-1**	glucose-1 attachment site
**Gly**	glycine
**GSH**	glutathione
**HFD**	high-fat diet
**HFDD**	high-fat diabetogenic diet
**His**	histidine
**HMGB1**	high mobility group box 1 protein
**HOMA-IR**	homeostasis model assessment of insulin resistance
**HPLC**	high-performance liquid chromatography
**AChEIs**	acetylcholinesterase inhibitors
**IDO**	indoleamine 2,3-dioxygenase
**Ile**	isoleucine
**IL**	interleukin
**IWQOL**	Impact of Weight on Quality of Life Questionnaire
**JAK2**	Janus kinase 2
**KA**	kainic acid
**KN**	kynurenine
**Leu**	leucine
**Lys**	lysine
**MCP-1**	monocyte chemoattractant protein 1
**MetHb**	methemoglobin
**MGO**	methylglyoxal
**MMP-13**	matrix metalloproteinase-13
**MPO**	myeloperoxidase
**NF-κB**	nuclear factor kappa B
**NFK**	N-formylkynurenine
**NGA**	non-glycated albumin
**NMDA**	N-methyl-D-aspartate receptor
**Nrf2**	nuclear factor erythroid 2-related factor 2
**P38 MAPK**	p38 mitogen-activated protein kinase
**PCs**	protein carbonyls
**PD**	Parkinson’s disease
**PEN**	pentosidine
**PGE1**	prostaglandin E1
**Pro**	proline
**RAGE**	receptor for advanced glycation end products
**RCS**	reactive carbonyl species
**RMSD**	root mean square deviation
**ROS**	reactive oxygen species
**RT-qPCR**	reverse transcription quantitative polymerase chain reaction
**S100B**	S100 calcium-binding protein B
**SI**	sucrase-isomaltase
**STAT**	signal transducer and activator of transcription
**Thr**	threonine
**Trp**	tryptophan
**Tyr**	tyrosine
**Val**	valine
**VES**	vesperlysine
