# Supplementary material for: Antiglycative Properties of Anti-Dementia Drugs—In Vitro, In Silico Studies and a Systematic Literature Review
Source: Antioxidants (Basel). 2025 Dec 16;14(12):1509. doi: 10.3390/antiox14121509 (PMC12729660; doi:10.3390/antiox14121509)
Supplement: Supplementary file 1 [file antioxidants-14-01509-s001.zip › antioxidants-4001393-supplementary.pdf]

**Table S1.** Complete results of the systematic review on the antiglycative properties of donepezil, rivastigmine, galantamine, memantine, lamotrigine, sodium valproate, and carbamazepine.

| Study design | End-points | |
|---|---|---|
| | ***In vitro* model** | |
| The study model was designed to assess the effect of memantine on AGEs-induced degradation of collagen II and aggrecan in chondrosarcoma cell lines (SW1353). AGEs were prepared using BSA, which was modified with MGO for 7 days. Then, SW1353 cells were exposed to memantine at concentrations of 5 and 10 μM for 24 hours and, after that time, to AGEs at a concentration of 100 μg/mL for 48 hours. | Memantine significantly protected SW1353 cells from AGEs-induced degradation of collagen II and aggrecan in a dose-dependent manner, which was associated with inhibition of matrix metalloproteinase-13 (MMP-13) and ADAM metalloproteinase with thrombospondin motifs 4 (ADAMTS-4) expression, as well as the inhibition of the activation of the Janus kinase 2/signal transducer and activator of transcription 1/interferon regulatory factor-1 (JAK2/STAT1/IRF-1) pathway. All changes were statistically significant at $p<0.01$. | [59] |
| | **Animal model** | |
| AGEs levels in the hippocampus and cerebral cortex were measured in male C57BL/6J mice fed a high-fat diet (HFD). Donepezil administration was initiated after 16 weeks of a high-fat diet (HFD), and treatment lasted 4 weeks in parallel with the continuation of the control diet (CD) or HFD. The mice were divided into four groups: control diet + saline (i.p.) (CD), HFD + saline (i.p.) (HFD), HFD + donepezil (HFDD, 3 mg/kg i.p.), control diet + donepezil (CDD, 3 mg/kg i.p.). AGEs levels were measured using ELISA kits. | A significant increase in AGEs was noticed in the HFD group compared to the control group (CD). Donepezil significantly reduced the elevated AGEs in the hippocampus and cerebral cortex in the HFDD group mice ($p<0.01$). In the control group, neither donepezil alone nor saline significantly affected AGEs levels. | [60] |
| The efficiency of donepezil in reducing various biomarkers of Alzheimer's disease (AD), including glycation parameters (Amyloid β 42, β-secretase, HbA1c), was analyzed using an animal model of streptozotocin (STZ)-induced hyperglycemia. Male Wistar rats were divided into three groups: a negative control group (no treatment), a positive control group (STZ, no treatment), and a group with hyperglycemia treated with donepezil (STZ+donepezil (10 mg/kg)). Drug administration was initiated on the 61st day of the experiment after induction of diabetes (STZ) at a dose of 42 mg/kg (i.p.); blood and brain glycation biomarkers were assessed afterward. | Donepezil ($p=0.07$) did not contribute to reducing blood glucose levels (after STZ induction). HbA1c levels in the positive control group (9.68±0.8%) were higher than in the negative control group (5.51±0.047%) ($p<0.05$), and the highest in the donepezil group (10.13±0.37%). Blood amyloid beta levels were significantly higher in all treatment groups compared to the positive control group ($p<0.05$) (donepezil: 546.16±5.82 pg/mL). In brain tissue, STZ significantly increased brain β-secretase levels, from 116.36±0.94 pg/mL (negative control group) to 862.03±8.38 pg/mL (positive control group, $p<0.05$). Donepezil treatment reduced beta-secretase levels to 187.05±1.94 pg/mL ($p<0.05$ vs. STZ). Amyloid beta 42 levels were significantly higher in the positive control group (778±15.85 pg/mL) compared to the negative control group (86±1.96 pg/mL) ($P<0.05$). Amyloid beta 42 levels were significantly lower in the donepezil group: 531.76±9.81 pg/mL) compared to the positive control group ($p<0.05$). | [61] |
| Sprague-Dawley rats were subjected to AD induction by intracerebral administration of amyloid beta (Aβ1–42) into the CA1 region of the hippocampus. Then, Lingguizhugan decoction (a Chinese herbal medicine | The Aβ1–42 level in the hippocampus was significantly lower in the donepezil-treated group (2.36±0.28 ng/mg tissue) compared to the AD-induced model group that did not receive this drug | [62] |

| | | |
|---|---|---|
| prepared according to a traditional recipe), donepezil (2 mg/kg), or distilled water was administered for 30 consecutive days. Administration: 30 days, twice daily. Behavioral tests assessing learning and memory were conducted to evaluate the treatment outcome. At the end of the experiment, Aβ1–42 levels in brain tissue were determined using ELISA kits. Lipoprotein-related receptor (LRP-1) and RAGE expression in the brain was analyzed using western blot, reverse transcription quantitative polymerase chain reaction (RT-qPCR), and immunohistochemical methods. | (4.17±0.35 ng/mg tissue; p<0.01). Donepezil significantly decreased RAGE expression in the hippocampus. Western blot analysis showed a 27% reduction in the expression in the donepezil group compared with the model group (p<0.05). In turn, the RAGE staining intensity in immunohistochemical studies was 0.52±0.08 in the donepezil group compared to 0.71±0.12 in the model group (p<0.05). Thus, donepezil showed a strong neuroprotective effect in the AD rat model. | |
| The study using an animal model of 2,4,6-trinitrobenzene sulfonic acid (TNBS)-induced colitis was to assess whether the antiglycative effect of galantamine involved the $\alpha$7 nAChR receptor. Wistar rats were divided into the following groups: control, TNBS, sulfasalazine-treated, galantamine-treated (10 mg/kg p.o.), methyl-likaconitine-treated (MLA; 5.6 mg/kg), and MLA+galantamine. The drugs were administered orally, once a day, for 11 days, and colitis was induced on day 8. | In the group of rats with TNBS-induced colitis, a more than two-fold increase in RAGE levels was observed compared to the control group (p<0.001). Galantamine treatment significantly reduced RAGE levels in the TNBS group by 48% compared to the TNBS group (p<0.001). The reduction in RAGE levels in the galantamine-treated group may be attributed to the activation of $\alpha$7 nAChR receptors, which inhibit inflammatory and oxidative processes by reducing the activity of NF-κB, the main transcription factor regulating RAGE expression. The level of HMGB1 (ligand for RAGE) is reduced, alleviating the inflammatory response. | [63] |
| The study was conducted in APP23 transgenic mice, a model of AD. The mice underwent carotid artery stenosis using ameroid clips, which led to gradual cerebral ischemia. The animals were divided into four groups: control (wild mice without intervention), APP23 mice without ischemia, APP23 mice with ischemia (CCH), and APP23 mice with ischemia treated with galantamine at a dose of 5 mg/kg daily p.o. Galantamine was administered from day 15, after carotid artery stenosis surgery (+/– 4 month of life), until euthanasia. The treatment lasted 1.5–8 months, and the mice's health was evaluated at 6 and 12 months. As part of the study, the levels of the protein glycation biomarkers, i.e., carboxymethyllysine (CML) and carboxymethylarginine (CMA), were measured. | The APP23+CCH group showed the highest levels of AGEs at 12-month follow-up, including CML in neurons and CMA in vessels and amyloid plaques (p<0.01 compared with the APP23 group without ischemia). Galantamine treatment significantly reduced the levels of these biomarkers—CML by approximately 30% and CMA by around 25%—in the APP23+CCH group compared to the untreated group (p<0.05). Galantamine is more effective when used longer (7.5 vs. 1.5 months of follow-up). This study confirmed that galantamine effectively limited damage associated with oxidative processes and glycation in the mice group with AD with chronic cerebral ischemia. | [64] |
| The study evaluated the effects of an alcoholic extract of *Laurus nobilis* leaves (bay leaf) on blood biochemical parameters and histological changes in the liver and kidney in sodium valproate-treated Wistar rats. The animals were divided into four groups (n=5): control (saline solution), *L. nobilis* extract (150 mg/kg bw), sodium valproate (500 mg/kg bw), and a combination of both substances. The rats were treated i.p. for 30 days. At the end of the experiment, blood samples were collected to analyze the levels of glucose, HbA1c, alanine aminotransferase (ALT), | In the group of rats treated with sodium valproate, the glucose level was 116.00±2.64 mg/dL, and that of HbA1c was 7.10±0.10 mg/dL, which was a significant increase (p<0.05) compared to the control group (glucose: 104.00±6.00 mg/dL; HbA1c: 6.73±0.21 mg/dL). The experiment showed that the alcoholic extract of *L. nobilis* leaves had a protective effect against the toxic impact of sodium valproate by reducing glycation parameters (glucose, HbA1c). | [65] |

| | | |
|---|---|---|
| aspartate aminotransferase (AST), creatinine, and urea, with liver and kidney tissues sampled for histological analysis. | | |
| The study assessed the effects of valproic acid on parameters related to glycation and pancreatic beta-cell function in a rat model of type 1 diabetes. Young Sprague-Dawley rats were divided into five groups: control (placebo), control receiving valproic acid (300 mg/kg/day), rats with STZ-induced diabetes, rats with diabetes treated with valproic acid at a dose of 150 mg/kg/day, and rats with diabetes treated with valproic acid at a dose of 300 mg/kg/day. Diabetes was induced through a single injection of STZ (75 mg/kg, i.p.), and valproic acid treatment lasted 3 weeks p.o. The plasma glucose and insulin levels, HbA1c, glucose tolerance (AUC), pancreatic islet morphology and function, beta-cell apoptosis (TUNEL test), and H3 histone acetylation were assessed. The analyses were performed using immunohistochemistry and biochemical tests (ELISA). | Plasma glucose levels increased from 116.06±4.06 mg/dL in the control group to 620.02±20.54 mg/dL in the STZ-induced diabetes group ($p<0.001$). Treatment with valproic acid at 300 mg/kg/day reduced glucose levels to 455.67±69.99 mg/dL ($p<0.01$ vs. diabetes). Glycated hemoglobin (%HbA1c) increased from 4.48±0.25 to 7.92±0.70 ($p<0.001$) in the STZ-induced diabetes group. Treatment with valproic acid at a dose of 150 mg/kg/day did not significantly change that indicator, while treatment with valproic acid at 300 mg/kg reduced HbA1c to 6.88±0.23, but the difference was not statistically significant. The study shows valproic acid protects pancreatic beta cells from apoptosis and promotes their regeneration in type 1 diabetes. The drug under study also improves histone H3 acetylation, which indicates an epigenetic mechanism. The results suggest the therapeutic potential of valproate in the treatment of diabetes. | [66] |
| The effect of long-term administration of *Uncaria rhynchophylla* (UR) extract on glycation parameters and inflammatory processes was assessed in a kainic acid (KA)-induced epilepsy model in rats. The animals were divided into four groups: a control group receiving phosphate buffer injection (PBS); KA injection (12 mg/kg, i.p.), KA injection and oral UR treatment (1 g/kg, 5 days/week for 6 weeks), and KA injection and oral valproic acid treatment (250 mg/kg, 5 days/week for 6 weeks) p.o. The animals were euthanized 42 days after KA administration. The assessment was performed utilizing histological examination (immunohistochemistry) of the hippocampus (CA1 region), analysis of protein expression [(S100B calcium-binding protein B, RAGE, metabotropic glutamate receptor 3 (mGluR3), monocyte chemoattractant protein 1 (MCP-1), chemokine receptor 2 (CCR-2)] using western blot analysis, observation of epileptic behavior, and electroencephalographic (EEG) recording. | Valproic acid treatment significantly reduced the S100B and RAGE levels in the hippocampus compared to the KA group. The S100B levels in the valproic acid group were 112.33±20.92 cells/field, significantly lower than in the KA group (260.67±34.89; $p<0.05$) and comparable to the UR group (129.17±19.38). RAGE expression was also significantly lower in the valproic acid-treated group (102.5±32.25 cells/field; $p<0.05$ vs. KA) and similar to UR. The results confirm the efficacy of valproic acid in reducing inflammation and glycation processes in the epilepsy model, with UR representing an effective natural alternative. | [67] |
| BALB/c mice were administered carbamazepine p.o. at a dose of 400 mg/kg for 4 days, followed by a dose of 800 mg/kg on day 5 of the experiment. Hepatic enzymes (ALT and AST), reduced glutathione (GSH) levels, markers of oxidative stress (protein carbonylation), expression of Toll-Like Receptor 4 (TLR4) and RAGE, and proinflammatory cytokines (IL-6, IL-23, IL-17) were analyzed. Additionally, the efficacy of TLR4 and RAGE blockade and IL-17 neutralization in reducing hepatotoxicity was tested. | Carbamazepine administration resulted in a significant increase (ALT and AST) vs. control ($p<0.05$). Hepatic GSH levels decreased significantly after 1.5, 3, 6, 12, and 24 hours from the last carbamazepine administration ($p<0.05$), accompanied by an increase in protein carbonylation ($p<0.05$). TLR4 expression increased significantly after 6 hours ($p<0.05$), and RAGE after 12 hours ($p<0.05$) compared to the baseline (0 h) or the control group. Administration of PGE1 | [68] |

| | | |
|---|---|---|
| Prostaglandin E1 (PGE1) was also administered to assess its potential protective properties. | contributed to the reduction of plasma levels of ALT, AST, IL-6, IL-23, and IL-17 ($p<0.05$), which suggests its protective effect in carbamazepine-induced hepatotoxicity. | |
| **Human model** | | |
| Twenty-one AD patients and ten subjects from the control group with similar metabolic profiles and comorbidities were treated with donepezil 10 mg/day, rivastigmine 9.5 mg/day, donepezil+memantine 10+20 mg/day). The drugs were administered orally. The patients were treated for at least 24 months. The levels of carbonyl stress biomarkers (AGEs), endothelial dysfunction, and inflammation were assessed. The concentration of AGEs in plasma was measured using the spectrofluorimetric method. | AGEs levels were 33% higher in AD patients compared to the control group, indicating increased endogenous glycation. Rivastigmine-treated patients had lower levels of AGEs compared to the other treatment groups ($p<0.05$) (however, the levels were higher than in the control group). In the other groups, AGEs levels remained higher than in the control group and did not differ significantly across treatment regimens. | [69] |
| The effect of rivastigmine on glycation was assessed. Two groups of patients participated in the study: patients with AD and comorbid diabetes and healthy volunteers of similar age without disease burden, who constituted the control group. Patients with AD received transdermal patches with rivastigmine at a dose of 4.6 mg for the first six weeks, then 9.5 mg daily until the end of the study (30 weeks). Serum AGEs levels were determined using ELISA or HPLC, while HbA1c levels were measured using standard biochemical tests. | The level of AGEs was significantly higher in AD patients compared with the control group (+32%; $p<0.05$). Treatment with rivastigmine resulted in a significant reduction in AGEs (−25%; $p=0.01$) after 30 weeks of the procedure. HbA1c levels were significantly higher in the study group (6.7±0.5%) compared to the control group (5.7±0.3%; $p=0.03$). After 30 weeks of treatment with rivastigmine, HbA1c levels in the study group decreased by 15% ($p<0.05$). | [70] |
| The study was designed to assess the efficacy and safety of lamotrigine for weight loss in obese adults. The single-center, randomized, double-blind, placebo-controlled study involved 40 persons (BMI: 30–39.9) divided into groups receiving lamotrigine p.o. (200 mg/day) or placebo for 26 weeks. Persons with type 1 diabetes, unstable type 2 diabetes, conditions affecting body weight, psychiatric disorders, and previous use of lamotrigine were excluded from the study. Changes in body weight (primary end-point), BMI, body fat percentage, HbA1c, lipid profile, quality of life (IWQOL), and satisfaction with the treatment were analyzed. | Lamotrigine contributed to a greater reduction in body weight ($p=0.0623$) and BMI ($p=0.0421$), with no significant differences in body fat or lipid profile. In terms of HbA1c, the difference between the lamotrigine group (0.1±0.3% increase) and the placebo group (0.0±0.3% increase) was statistically insignificant ($p=0.9093$), indicating no effect of lamotrigine on glycemic regulation in the study group. | [71] |
| The cross-sectional study included patients with idiopathic generalized tonic–clonic epilepsy treated with valproic acid (400–1750mg/day, n=22) p.o. for at least one year, treated with lamotrigine (100–500mg/day, n=22) p.o. over the same period, newly diagnosed or untreated patients (n=22), and healthy controls (n=22). Metabolic parameters such as fasting glucose, hemoglobin HbA1c, fasting insulin, lipid profile, insulin resistance index (HOMA-IR), and asprosin levels, which is a hormone (adipokine) secreted by white adipose tissue that regulates glucose metabolism and appetite, were assessed. Asprosin stimulates the liver to release glucose into the bloodstream and acts on the hypothalamus to | The patients treated with valproic acid had significantly higher fasting glucose levels (mean 107.8±11.6 mg/dL) compared to the control group (91.4±8.9 mg/dL; $p<0.01$), the lamotrigine group (95.3±9.1 mg/dL; $p<0.05$) and the newly diagnosed or untreated group (94.8±8.7 mg/dL; $p<0.05$). A similar trend was observed for HbA1c, where the mean level in the valproic acid-treated group was 6.2±0.4%, which was significantly higher compared with the control group (5.5±0.3%; $p<0.01$) and the lamotrigine-treated group (5.7±0.4%; $p<0.05$). Higher HbA1c and fasting glucose levels in the valproic acid-treated group combined with increased insulin levels (12.8±3.6 μIU/mL vs. 8.3±2.4 | [72] |

| | | |
|---|---|---|
| increase the feeling of hunger. Elevated hormone levels are associated with insulin resistance, obesity, and metabolic disorders. Blood was collected on an empty stomach, and analyses were performed using standard methods (ELISA for asprosin). | μIU/mL in the control group; $p<0.05$) may indicate increased protein glycation as a result of valproic acid therapy. Glycation levels in the lamotrigine-treated group were comparable to that in the control group. | |
| The study assessed the effect of antiepileptic drugs on the levels of the C-reactive protein (CRP) and HbA1c, as well as the estimated glomerular filtration rate (eGFR). The study included 125 patients with drug-resistant epilepsy, 46 of whom were male, aged 47 on average (age range 23–85). In the study group, patients were administered 1 to 5 different drugs for many years, and 18 patients used monotherapy. The most commonly used drugs were sodium valproate (N=50, mean dose 1000–2000 mg/day), lamotrigine (N=48, 100–400 mg/day), oxcarbazepine (N=46, 600–2400 mg/day), carbamazepine (N=37, 400–1600 mg/day), phenytoin (N=32, 200–400 mg/day), and levetiracetam (N=31, 500–3000 mg/day). The correlations between drug use and altered biomarker levels were assessed using the multivariate regression analysis. | With regard to hemoglobin HbA1c, lamotrigine administration revealed a trend toward a 2% reduction, but this difference was not statistically significant ($p=0.11$). The use of sodium valproate ($p=0.70$) and carbamazepine ($p=0.24$) did not reveal significant changes in HbA1c levels. However, sodium valproate reduced CRP by 55% ($p=0.001$) and improved eGFR by 10% ($p=0.018$), and phenytoin reduced HbA1c by 4% ($p=0.004$). Lamotrigine reduced eGFR by 13% ($p=0.001$). | [73] |
| The study assessed the effect of age and biochemical parameters in humans on the binding of carbamazepine to serum proteins. It focused on glycated and non-glycated hemoglobin (NGA). *In vitro* and *in vivo* analyses were performed on a group of 66 patients (aged 4–83) with epilepsy or neuralgia treated p.o. for many years (reaching a steady state of the drug). Correlations between carbamazepine free fraction (CBZ-FF%) and glycated and non-glycated albumin and age were assessed. | With age, the level of glycated albumin increases ($r=0.666$; $p<0.001$), and the level of non-glycated albumin decreases ($r=–0.459$; $p<0.001$), which contributes to the increase in CBZ-FF% ($r=0.992$; $p<0.001$). In the elderly, the highest level of glycated albumin (17.3%) and the lowest level of non-glycated albumin (3.3 g/dL) resulted in the highest CBZ-FF% (31.1%; $p<0.001$). Glycation, which increases with age, reduces the availability of non-glycated albumin, increasing the toxicity of carbamazepine. However, carbamazepine alone does not appear to modify albumin glycation. | [74] |
| This study was designed to assess the effect of chronic hyperglycemia on valproic acid binding to serum proteins. Two groups participated in the study: insulin-dependent diabetes (IDD) patients and healthy controls. In the diabetic group (n=62), the mean albumin concentration was 38.7±1.9 g/L, and the mean HbA1c level was 4.1±2.1%. In the control group (n=22), the mean albumin concentration was 38.6±2.2 g/L, and the HbA1c level was 1.1±0.5%. Valproic acid (100 mg/L) binding to serum proteins was assessed by equilibrium dialysis, and its concentration was measured by immunopolarization of fluorescence. | Valproic acid binding was significantly lower in diabetes patients (75.2±6.7%) compared to the control group (80.7±4.9%), with the difference being statistically significant ($p<0.001$). However, no significant correlation was found between the HbA1c percentage and valproic acid binding. The results suggest that reduced valproic acid binding in the serum of diabetics is related to diabetes-specific albumin modifications independent of glycation. | [75] |
| The study involved 23 patients (both sexes, middle-aged) with epilepsy who were treated with carbamazepine (serum drug concentrations were within the therapeutic range) p.o. for more than two years. The control group consisted of healthy volunteers (aged 17–35). Hemolyzed blood samples were analyzed to assess hemoglobin fractions for | In the hemolysates of patients treated with carbamazepine, the level of Hb ASSG was 2–7% of total hemoglobin. It was significantly higher compared to the control group, where Hb ASSG was not detected ($p<0.01$). In the study group, the methemoglobin (MetHb) level was 8.5%±1.5%, which was significantly higher compared to that in | [76] |

| | |
|---|---|
| glutathione adduct of hemoglobin (Hb ASSG) using ion exchange chromatography and electrophoresis. | the control group (3%±0.8%; $p<0.001$). The content of thiols (SH) in Hb ASSG was significantly lower (0–1 SH group; $p<0.05$) compared to control hemoglobin (2 SH groups). A significant correlation was found between the level of Hb ASSG and MetHb ($r=0.8$; $p<0.01$), indicating oxidative stress, which increases hemoglobin glycation in erythrocytes of patients undergoing long-term carbamazepine therapy. |
